# Acupuncture for post-cesarean pain and gastrointestinal function recovery: a meta-analysis and systematic review

**DOI:** 10.3389/fmed.2025.1583898

**Published:** 2025-06-18

**Authors:** Hejing Liu, Kehan Xing, Cai Liao, Jiapei Wang, Chenglin Tang, Siqin Huang

**Affiliations:** College of Traditional Chinese Medicine, Chongqing Medical University, Chongqing, China

**Keywords:** acupuncture, cesarean, pain, gastrointestinal function, meta-analysis

## Abstract

**Objective:**

Postoperative pain and gastrointestinal dysfunction are common complications after cesarean section, significantly affecting maternal recovery and quality of life. Acupuncture has recently shown promise in alleviating postoperative pain and promoting the recovery of gastrointestinal function. This study aimed to assess the effectiveness of acupuncture in relieving postoperative pain and improving gastrointestinal function after cesarean section via a meta-analysis.

**Materials and methods:**

A comprehensive search for randomized controlled trials (RCTs) examining acupuncture for post-cesarean pain and gastrointestinal function was conducted across multiple databases, covering literature up to 1 February 2025. Data analysis was conducted using Stata 15.

**Result:**

A total of 26 studies involving 2,641 patients were included. This meta-analysis evaluated the effects of acupuncture on postoperative pain, bowel sound recovery, and time to first flatus. The results demonstrated that acupuncture significantly reduced VAS at 6 h (SMD = −0.36, 95%CI [−0.65, −0.07]), 12 h (SMD = −1.23, 95%CI [−1.81, −0.64]), 24 h (SMD = −1.06, 95%CI [−1.56, −0.56]), and 48 h (SMD = −0.96, 95%CI [−1.76, −0.17]). Additionally, acupuncture significantly shortened bowel sound recovery time (SMD = −2.26, 95% CI [−2.97, −1.54]) and anal exhaust time (SMD = −2.41, 95% CI [−3.21, −1.61]). Subgroup analysis revealed that conventional acupuncture was effective across the majority of outcomes, while electroacupuncture showed comparatively weaker effects at certain time points. However, the presence of substantial heterogeneity (I^2^ values above 90%), along with variations in study quality and acupuncture protocols, could limit the accuracy and generalizability of the findings.

**Conclusion:**

This meta-analysis suggests that acupuncture may effectively relieve pain and improve gastrointestinal function after cesarean section. The study’s results showed significant improvements in pain scores and gastrointestinal recovery indicators, including bowel sound recovery time and anal exhaust time. However, the results should be interpreted with caution due to the high degree of heterogeneity and variability in study quality and acupuncture protocols. Further high-quality, large-scale RCTs are needed to validate these findings.

**Systematic review registration:**

https://www.crd.york.ac.uk/PROSPERO/view/CRD42025638696.

## Introduction

Cesarean section is one of the most commonly performed obstetric and gynecological procedures worldwide. While it plays a vital role in ensuring the safety of mothers and babies, postoperative pain and gastrointestinal dysfunction remain significant challenges that adversely affect maternal recovery (1, 2). According to the World Health Organization, the global cesarean section rate has increased from approximately 7% in 1990 to 21% today, and this number is projected to continue increasing through 2030. Estimated future rates include 63% in East Asia, 54% in Latin America and the Caribbean, 50% in West Asia, 48% in North Africa, 47% in Southern Europe, and 45% in Australia and New Zealand (3). As cesarean section rates continue to rise year by year, effective management of postoperative pain and gastrointestinal recovery is becoming increasingly critical (4). Postoperative pain affects not only the physical comfort of the mother but may also lead to psychological issues such as anxiety and depression. Inadequate pain management may hinder mother–infant bonding and interfere with successful breastfeeding initiation (5, 6). In addition, postoperative gastrointestinal disturbances—such as nausea, vomiting, and constipation—are commonly experienced by women following a cesarean section. These issues are often associated with the side effects of anesthesia (7), surgical trauma, and restricted maternal mobility during the postoperative period (8). The development of post-cesarean pain is multifactorial, involving surgical trauma, uterine contraction, abdominal incision healing, the use of anesthetics and analgesics, as well as individual patient differences (9, 10). Pain typically manifests as localized incisional pain and abdominal pain due to uterine contractions. Beyond discomfort, such pain may impair daily functioning and disrupt mother–infant interaction (11). Traditional pharmacological analgesic measures, although capable of relieving pain to some extent, are often accompanied by side effects such as constipation, nausea, and vomiting. Gastrointestinal dysfunction after cesarean section, such as nausea, vomiting, bloating, and constipation, is also common. The inhibitory effect of anesthetic drugs, particularly those used in general anesthesia, on gastrointestinal function is a major cause of postoperative gastrointestinal dysfunction (12). Postoperative mothers often suffer from gastrointestinal discomfort due to restricted activities, delayed intestinal peristalsis, suboptimal dietary practices, and other factors. Poor recovery of gastrointestinal function affects not only the overall health of the mother but may also affect the speed of her recovery and extend hospital stays (13). Given these challenges, it is particularly important to find a safe and effective non-pharmacological treatment with fewer side effects.

In recent years, acupuncture, a traditional Chinese medicine treatment, has gained widespread attention for its unique efficacy in relieving pain and improving gastrointestinal function (14). By stimulating specific acupoints to regulate the flow of qi and blood and balancing yin and yang, acupuncture can promote the body’s self-regulation and improve various symptoms. Especially after cesarean section, acupuncture is believed to help mothers recover better by relieving pain, promoting blood circulation, regulating the nervous system, and restoring gastrointestinal function (15). Although several studies have been conducted to investigate the role of acupuncture in the recovery of pain and gastrointestinal function after cesarean section, the majority of the studies still have some limitations (16, 17). For example, the acupuncture protocols (including point selection, treatment duration, and treatment frequency) in the studies varied widely, resulting in poorly comparable findings, and with the gradual increase in the number of RCTs of acupuncture interventions for post-cesarean section pain and gastrointestinal function recovery (18). Therefore, it is necessary to conduct a systematic evaluation and meta-analysis to comprehensively sort out and quantitatively analyze the existing evidence on acupuncture for the treatment of postoperative pain and gastrointestinal function recovery after cesarean delivery. This study aims to clarify the efficacy and safety of acupuncture, enhance its clinical value in postpartum rehabilitation, and provide a scientific basis for developing a pathway for postpartum pain management and rapid recovery, thereby promoting maternal and infant health and reducing healthcare burdens.

## Materials and methods

The systematic review was supported by the online PROSPERO International Prospective Register of Systematic Reviews (19) of the National Institute for Health Research (CRD42025638696).

### Literature search

This study was conducted through a search of CNKI, Wan Fang, VIP, PubMed, Embase, Cochrane Library, and Web of Science from database inception to 1 February 2025, using the search terms “acupuncture” and “cesarean section.” A specific search strategy is described in the Supplementary Table S1. CNKI, Wan Fang, and VIP are the primary academic databases in China, encompassing numerous peer-reviewed clinical studies, particularly randomized controlled trials (RCTs) related to traditional Chinese medicine therapies, such as acupuncture.

### Inclusion and exclusion criteria

The inclusion criteria for this study were that participants were post-cesarean section mothers aged >18 years without severe cardiovascular disease, hepatic or renal dysfunction, or other major comorbidities. The experimental group was treated with acupuncture, and the control group was treated with sham acupuncture; the primary endpoints were pain scores, time to recovery of bowel sounds, and time to anal defecation, and the secondary endpoint was the length of hospital stay. Only randomized controlled studies were considered for inclusion in this study.

The exclusion criteria for this study included case reports, case series, expert opinions, reviews and review articles, studies without explicit acupuncture interventions, unpublished studies or conference abstracts, non-peer-reviewed research articles, and maternal comorbidities with underlying chronic conditions that significantly affect pain or gastrointestinal function, such as chronic pain syndromes, functional gastrointestinal dysfunction (irritable bowel syndrome and chronic constipation), history of gastrointestinal surgery, diabetes mellitus, and rheumatoid arthritis.

### Literature screening and data extraction

Literature screening was conducted using EndNote 21 software to ensure that included studies met the inclusion criteria for this study and to exclude studies that did not. Two independent researchers conducted the literature screening process. In cases of disagreement, a third researcher was consulted to resolve the dispute. Data were extracted independently by two researchers using a pre-designed standardized form. The extracted information included (1) basic study details—first author and year of publication; (2) population characteristics—sample size, average age, number of pregnancies and births; (4) interventions—type of acupuncture (body acupuncture, electroacupuncture, auricular acupuncture, or acupoint placement), frequency, and duration of intervention; (5) control group details; and (6) outcomes. In cases of disagreement between two researchers during the extraction process, the issue was first discussed and resolved; if necessary, a third researcher was consulted to make a final decision. For important missing or unclear information, attempts were made to contact the original authors to obtain additional data.

### Risk of bias assessment

In this study, quality evaluation was assessed using the ROB (20). The ROB tool is used to assess the risk of bias in randomized controlled trials (RCTs). It consists of seven dimensions: random sequence generation, allocation concealment, blinding implementation, data completeness, selective reporting, other sources of bias, and study funding. The risk of bias for each aspect was categorized as high, unclear, or low. Specifically, studies were rated as low bias if the methodology was clearly described and compliant, high bias if the methodology was opaque or likely to lead to systematic error, and moderate bias if no specific bias could be identified. Additionally, each domain was assessed in conjunction with the trial’s design, execution, and reporting quality. The application of this tool helps ensure the quality and reliability of the included studies, thus improving the credibility of the results of this study. All assessments were conducted by two independent reviewers. In cases of disagreement, a resolution was reached through discussion.

### GRADE assessment

In this study, the quality of evidence was assessed using the GRADE system (21). The GRADE (Grading of Recommendations Assessment, Development, and Evaluation) system classifies evidence into four grades: high, medium, low, and very low quality. The rating process considers factors such as study design, risk of bias, directness, consistency, precision, and reporting bias. High-quality evidence typically comes from well-conducted randomized controlled trials (RCTs) or exceptionally rigorous observational studies with a low risk of bias, consistent findings, and directly applicable results. Moderate-quality evidence suggests that the study may have some limitations, such as a risk of bias, indirectness, or imprecision; however, the overall conclusions are likely to remain reliable. Low-quality evidence indicates that the study has significant methodological flaws or other issues that reduce confidence in the findings. Very low-quality evidence typically comes from studies with serious limitations, and the conclusions are highly uncertain, requiring further research to confirm the results.

### Data analysis

Stata 15.0 was used for data analysis in this study. For continuous variables (pain scores, time to bowel sound recovery, time to anal defecation, and length of hospital stay), standardized mean differences (SMDs) with 95% confidence intervals (CIs) were used for combined analyses. Heterogeneity was tested using the *Q*-test and *I*^2^. When *I*^2^ was greater than 50%, a random effects model was used for analysis, and sensitivity analysis was performed to explore the sources of heterogeneity.

When *I*^2^ was less than 50%, the fixed effects model was used for analysis. Publication bias was assessed using the Egger test, and a *p*-value of less than 0.05 was considered indicative of a more likely publication bias. This study was analyzed in subgroups according to the type of acupuncture.

## Results

### Literature search results

**Figure 1:**
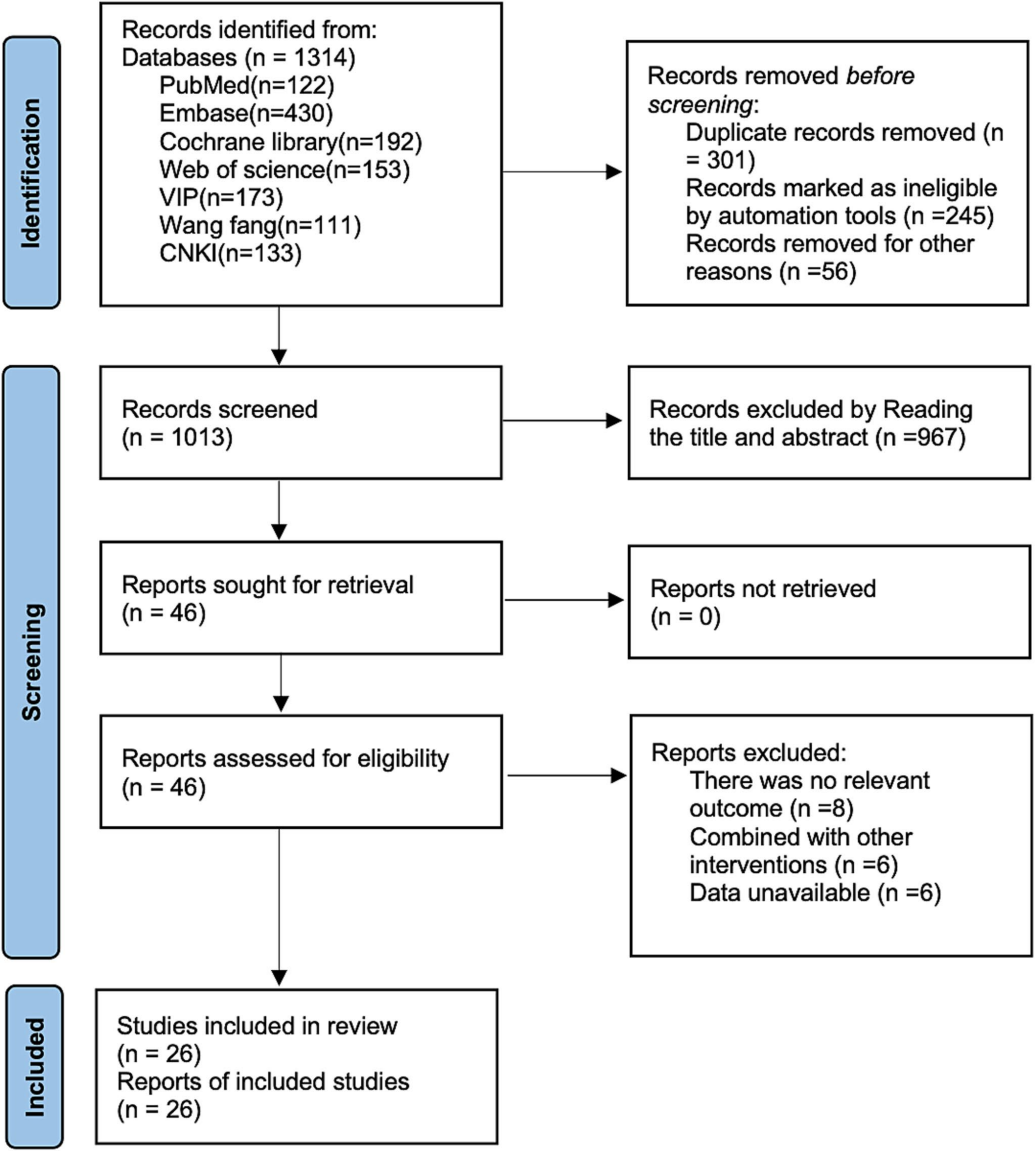
Literature search flowchart.

A total of 26 randomized controlled trials (22–47) were included following an initial search of CNKI (*n* = 133), WanFang (*n* = 111), VIP (*n* = 173), PubMed (*n* = 122), Embase (*n* = 430), Cochrane Library (*n* = 1,920), and Web of Science (*n* = 153). After removing duplicates (*n* = 301), screening titles and abstracts (*n* = 967), and reviewing full texts (*n* = 20), 26 RCTs were ultimately included. The flowchart of the literature retrieval process is shown in Figure 1.

### Basic characteristics of literature

As shown in Table 1, a total of 26 studies involving 2,641 patients were included in this study, with an age range of 23–34.1 years. The ASA classification was I-III. Acupuncture modalities included traditional acupuncture, warm acupuncture, electroacupuncture, laser acupuncture, fine needle pricking, and auricular acupuncture.

**Table 1:** Basic characteristics table.

Study	Year	Sample size		Mean age (years)		ASA score	Pregnancy times	Comorbidity	Intervention		Frequency	Duration of each treatment	Outcomes
		EG	CG	EG	CG				EG	CG			
Brase	2022	38	42	32	32	ASA I-III	Primipara/Multipara	NR	Laser acupuncture: 830 nm	Placebo	Twice/day	30 min	F1; F2
Gamesman	2015	28	28	28	26.9	ASAI/III	Primipara/Multipara	NR	Acupuncture: P6 and LI4	Placebo	Twice/day	30 min	F1
Jin	2023	106	52	30.45	31.19	NR	NR	NR	Electroacupuncture: 2/20/100HZ	Placebo	Three times/ day	20 min	F1; F3
Mazda	2018	20	18	34.3	33.7	NR	NR	None	Acupuncture: P6 and LI4	Placebo	Three times/ day	20 min	F1
Usichenko	2022	60	60	31	31	ASAII/III	Primipara/Multipara	None	Acupuncture: LI4	Placebo	Once/day	20 min	F1; F2
WU	2009	40	20	30.9	30.8	ASAI/II	Primipara	None	Acupuncture: LI4; Electroacupuncture: 2HZ	Placebo	Once/day	15 min	F1
YP Yi	2014	40	32	25.2	26.5	ASAI/II	NR	NR	Acupuncture: LI4 + TCM	Placebo	Three times/ day	30 min	F4; F5; F6
Y He	2022	52	52	28.36	27.42	NR	NR	NR	Acupuncture: LI4	Placebo	NR	20 min	F1
L Liu	2023	30	30	29.4	30.3	NR	NR	NR	prick needling + TCM	Placebo	Once/day	NR	F1; F5; F6
YJ Zhang	2023	42	42	31.22	31.88	NR	NR	NR	Electroacupuncture + TCM	Placebo	Twice/day	30 min	F5; F6
RY Zhang	2022	35	44	30.7	30.7	ASAI/II	NR	NR	prick needling	Placebo			F1
X Cheng	2017	34	34	27.4	27.7	NR	NR	NR	Warm acupuncture	Placebo	Twice/day	NR	F5; F6
TZ Li	2017	30	30	30	32	ASAI/II	NR	NR	Electroacupuncture: 2HZ	Placebo	Twice/day	NR	F1
XQ Li	2023	50	50	26.46	26.69	NR	NR	NR	Prick needling	Placebo	Twice/day	NR	F1; F5; F6
KM Yang	2019	80	80	30.9	31.2	NR	NR	NR	Acupuncture: P6 and LI4	Placebo	NR	20 min	F5; F6
GQ Yang	2010	110	110	25.8	25.8	NR	NR	NR	Acupuncture: LI4 + TCM	Placebo	NR	20 min	F5; F6
GY Yang	2019	60	60	28	28	NR	NR	NR	Auricular acupuncture	Placebo	NR	20 min	F6
T Liu	2023	36	36	28.45	28.32	NR	NR	NR	Auricular acupuncture	Placebo	NR	30 min	F5; F6
W Pan	2024	50	50	25.84	25.61	NR	NR	NR	Pestle acupuncture	Placebo	NR	30 min	F1
L Wang	2023	40	40	27.48	38.01	NR	NR	NR	Pestle acupuncture	Placebo	NR	30 min	F5; F6
CF Liao	2019	29	31	25.22	25.69	NR	Primipara/Multipara	None	Electroacupuncture: 2HZ	Placebo	NR	30 min	F5; F6
MJ Hu	2024	50	50	31.6	31.2	NR	Primipara/Multipara	None	prick needling	Placebo	Twice/day	20 min	F5; F6
LP Jiang	2012	130	126	28	27	NR	NR	NR	Electroacupuncture: 2HZ	Placebo	Twice/day	20 min	F5; F6
LY Xu	2021	50	50	27.48	27.64	ASAI/II	NR	NR	Electroacupuncture: 2HZ	Placebo	Twice/day	45 min	F1
Y Ma	2024	35	35	29.51	30.46	NR	NR	NR	Acupuncture: P6 and LI4	Placebo	Twice/day	45 min	F6
ZX Lu	2017	82	82	23–44		ASAI/II	NR	NR	Warm acupuncture	Placebo	Twice/day	30 min	F5; F6

EG: experiment group; CG: control group; ASA: American Society of Anesthesiologists; NR: not report; TCM: Traditional Chinese medicine; F1: pain scores; F2: Length of stay; F3: Fentanyl consumption; F4: Time for bloating to disappear; F5: Bowel sound recovery time; F6: Anal exhaust time.

### Risk of bias and GRADE result

Five of the studies (28, 30, 32, 43, 44) included in the current round did not clearly report the blinding method and were therefore assessed as having an unclear risk of bias. Three studies (31, 43, 44) explicitly lacked information on blinding and were assessed as high-risk. Specific risk of bias scores are described in Supplementary Figures S1, S2. The GRADE rating for this analysis is presented in the Supplementary Table S2.

### Meta-analysis results

#### 6-h pain scores

**Figure 2:**
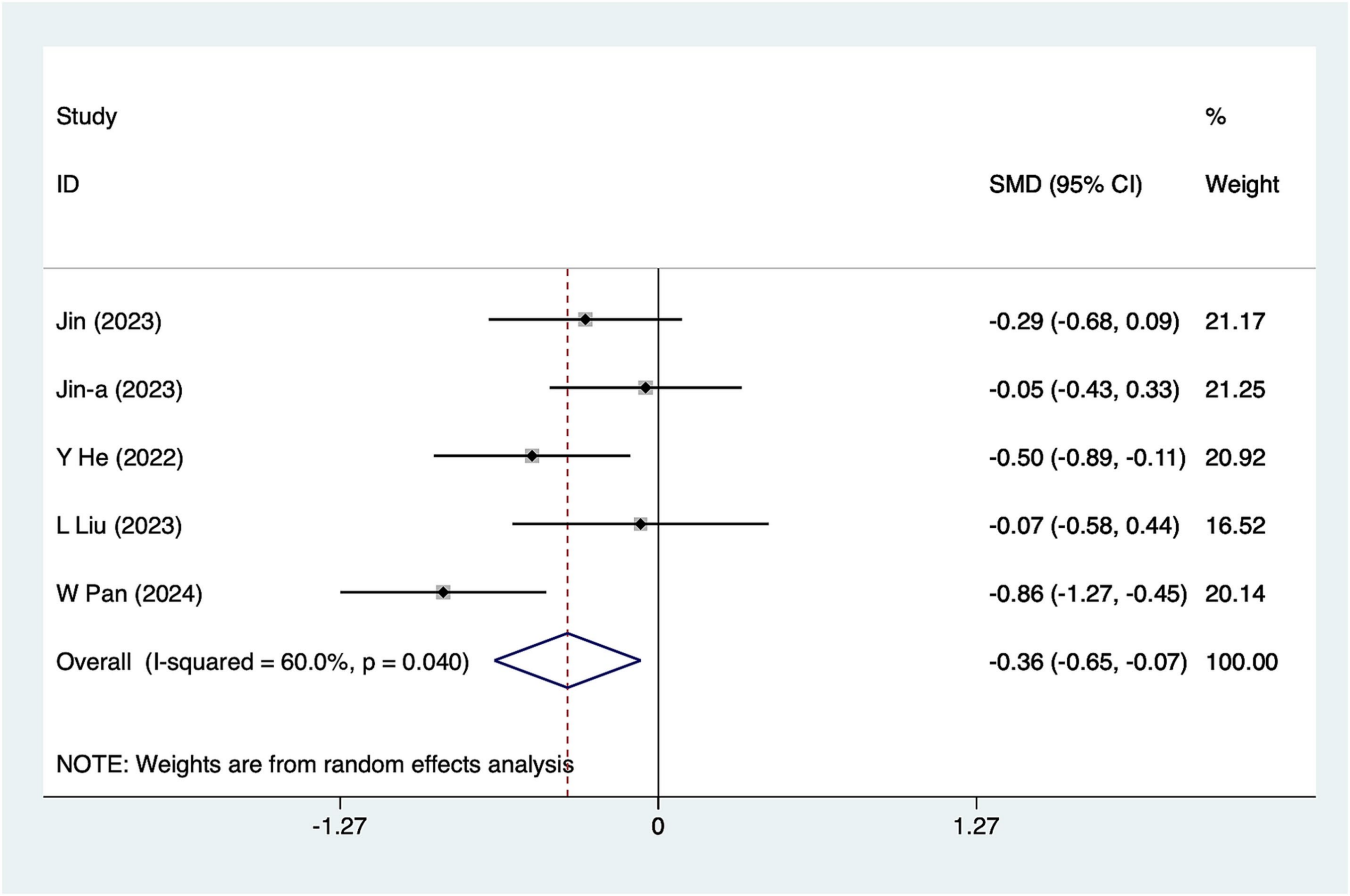
A 6-h pain score meta-analysis forest plot. The coordinate value corresponding to the box represents the difference in response rate between the experimental group and the control group for each study. The value range covered by the horizontal line represents the confidence interval for each study. The bottom diamond represents the aggregated effect.

Five studies reported 6-h pain scores. Due to moderate heterogeneity (*I*^2^ = 60%, *p* = 0.04), a random-effects model was used for analysis. The results (Figure 2) indicated that acupuncture significantly reduced 6-h pain scores [SMD = −0.36, 95% CI (−0.65, −0.07)]. Subgroup analyses based on different acupuncture modalities (Table 2) showed that electroacupuncture was ineffective for 6-h pain scores. Given the considerable heterogeneity, sensitivity analyses were conducted using a leave-one-out (literature-by-exclusion) approach (Supplementary Figure S3). The results suggested minimal sensitivity, suggesting that the analyses were stable.

**Table 2:** Results of subgroup analyses.

Outcomes	Subgroup	No of study	Heterogeneity		SMD (95%CI)	*P*
			*I*^2^ (%)	*P*		
6 h-pain scores	Electroacupuncture	2	0	0.386	−0.17 (−0.44, 0.10)	0.121
	Acupuncture	1	NR	NR	−0.5 (−0.89, −0.11)	0.001
	Acupuncture + TCM	1	NR	NR	−0.07 (−0.58, 0.44)	0.235
	Pestle acupuncture	1	NR	NR	−0.86 (−1.27, −0.45)	0.029
12 h-pain scores	Electroacupuncture	3	86.8	0.001	−2.09 (−2.93, −1.25)	0.001
	Acupuncture	3	87.8	0.0001	−0.63 (−1.45, 0.19)	0.349
	Acupuncture + TCM	1	NR	NR	−0.61 (−1.06, −0.15)	0.002
	Pestle acupuncture	1	NR	NR	−1.10 (−1.52, −0.68)	0.010
24 h-pain scores	Laser acupuncture	1	NR	NR	−0.11 (−0.55,0.33)	0.431
	Electroacupuncture	5	79.3	0.001	−0.59 (−1.10, −0.08)	0.001
	Acupuncture	5	94.5	0.0001	−1.94 (−2.99, −0.89)	0.031
	Acupuncture + TCM	2	0	0.810	−0.32 (−0.66, 0.02)	0.340
	Pestle acupuncture	1	NR	NR	−1.55 (−2.00, −1.10)	0.001
48 h-pain scores	Laser acupuncture	1	NR	NR	0.15 (−0.29, 0.59)	0.130
	Electroacupuncture	4	96.3	0.0001	−1.97 (−3.32, −0.63)	0.001
	Acupuncture	1	NR	NR	0.62 (0.09, 1.16)	0.001
	Acupuncture + TCM	2	89.3	0.002	−0.54 (−1.62, 0.54)	0.230
	Pestle acupuncture	1	NR	NR	−0.51 (−0.91, −0.11)	0.010
	Acupuncture	2	74	0.05	−0.26 (−0.79, 0.26)	0.160
	Auricular acupuncture	1	NR	NR	−0.39 (−0.75, −0.03)	0.001
Bowel sound recovery time	Acupuncture + TCM	3	94.2	0.001	−2.77 (−4.12, −1.42)	0.011
	Electroacupuncture + TCM	1	NR	NR	−2.78 (−3.38, −2.17)	0.001
	Warm acupuncture	2	98.1	0.001	−4.55 (−8.27, −0.84)	0.003
	Acupuncture	3	78	0.011	−1.06 (−1.54, −0.58)	0.001
	Auricular acupuncture	1	NR	NR	−0.92 (−1.40, −0.43)	0.020
	Pestle acupuncture	1	NR	NR	−1.34 (−1.83, −0.86)	0.001
	Electroacupuncture	2	98.1	0.001	−1.99 (−4.30, 0.33)	0.221
Anal exhaust time	Acupuncture + TCM	3	83.1	0.003	−2.97 (−3.82, −2.13)	0.001
	Electroacupuncture + TCM	1	NR	NR	−2.81 (−3.42, −2.20)	0.021
	Warm acupuncture	2	99.4	0.001	−6.05 (−14.48, 2.39)	0.231
	Acupuncture	4	91.0	0.001	−1.18 (−1.89, −0.47)	0.002
	Auricular acupuncture	2	78.3	0.032	−0.78 (−1.45, −0.12)	0.034
	Pestle acupuncture	1	NR	NR	−1.10 (−1.58, −0.63)	0.021
	Electroacupuncture	2	99.2	0.001	−2.58 (−6.80, 1.09)	0.435

#### 12-h pain scores

**Figure 3:**
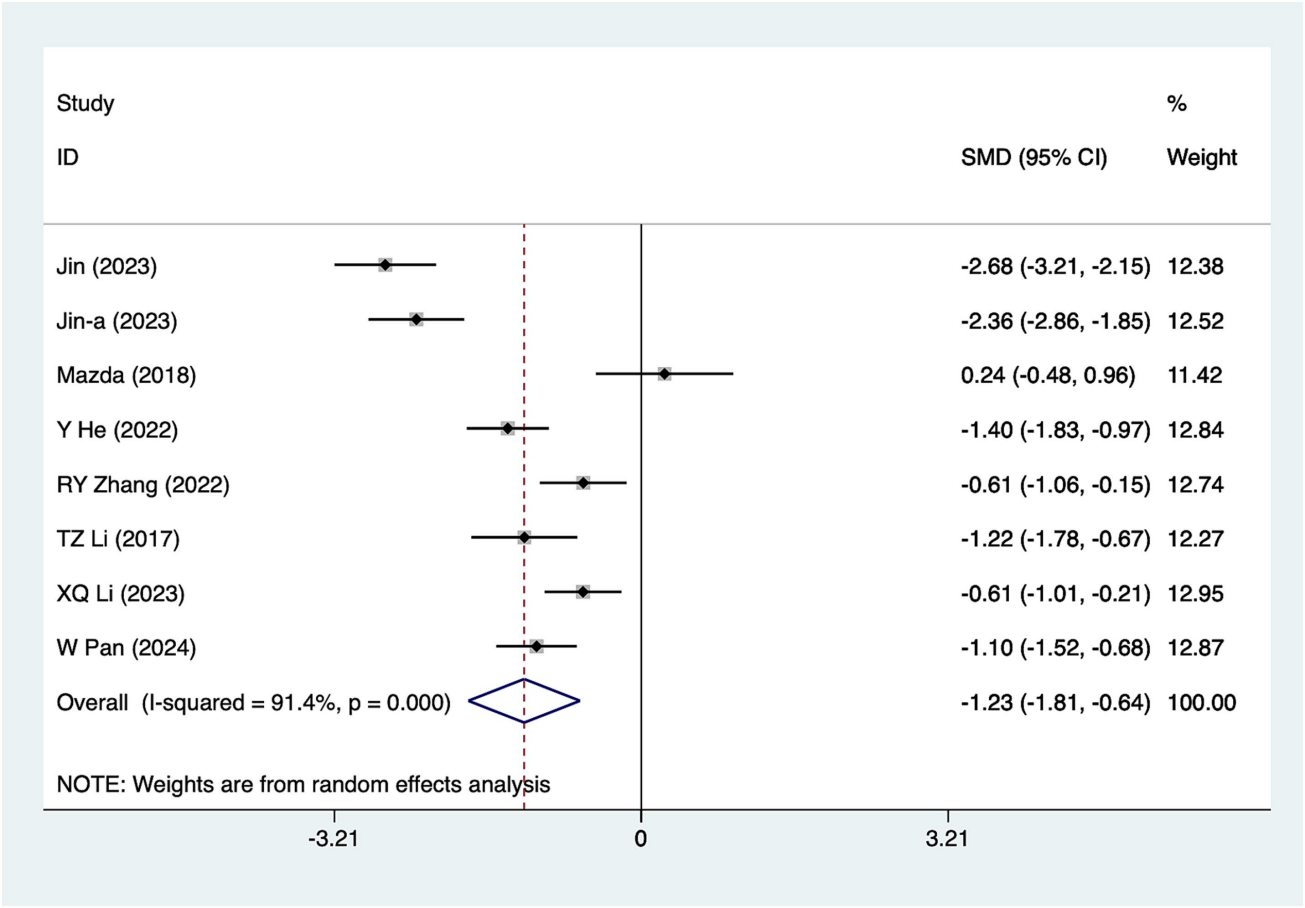
A 12-h pain score meta-analysis forest plot. The coordinate value for the box represents the difference in response rate between the experimental group and the control group for each study, while the range of values covered by the horizontal line represents the confidence interval for each study. The bottom diamond represents the aggregated effect.

A total of eight studies reported 12-h pain scores. Due to substantial heterogeneity (*I*^2^ = 91.4%, *p* = 0.001), a random-effects model was used for analysis. The results (Figure 3) suggested that acupuncture significantly reduced 12-h pain scores [SMD = -1.23, 95%CI (−1.81, −0.64)]. Subgroup analyses based on different acupuncture modalities (Table 2) showed that electroacupuncture was effective in reducing 12-h pain scores, whereas conventional acupuncture was not. Due to the considerable heterogeneity, sensitivity analyses were conducted using a leave-one-out (literature-by-exclusion) approach (Supplementary Figure S4). The results suggested minimal sensitivity, indicating that the analyses were stable.

#### 24-h pain scores

**Figure 4:**
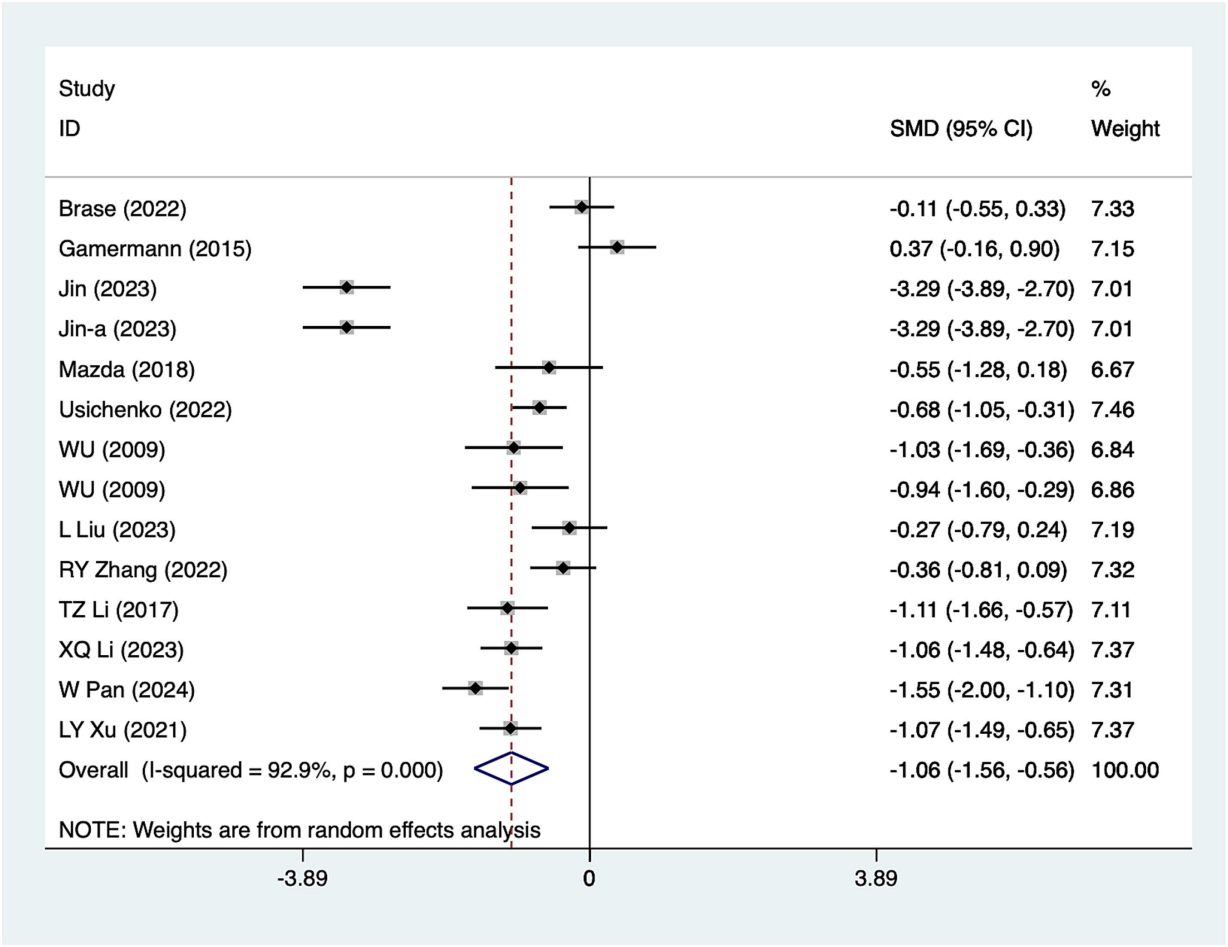
A 24-h pain score meta-analysis forest plot. The coordinate value corresponding to the box represents the difference in response rate between the experimental group and the control group for each study, while the range of values covered by the horizontal line represents the confidence interval for each study. The bottom diamond represents the aggregated effect.

A total of 14 studies reported 24-h pain scores. Due to substantial heterogeneity (*I*^2^ = 92.9%, *p* = 0.001), a random-effects model was used for analysis. The results (Figure 4) suggested that acupuncture significantly reduced 24-h pain scores [SMD = –1.06, 95%CI (−1.56, −0.56)]. Subgroup analyses based on different acupuncture modalities (Table 2) showed that both electroacupuncture and conventional acupuncture were effective in reducing 24-h pain scores. Given the considerable heterogeneity, sensitivity analyses were conducted using a leave-one-out (literature-by-exclusion) approach (Supplementary Figure S5). The results indicated minimal sensitivity, suggesting that the analyses were robust and stable.

#### 48-h pain scores

**Figure 5:**
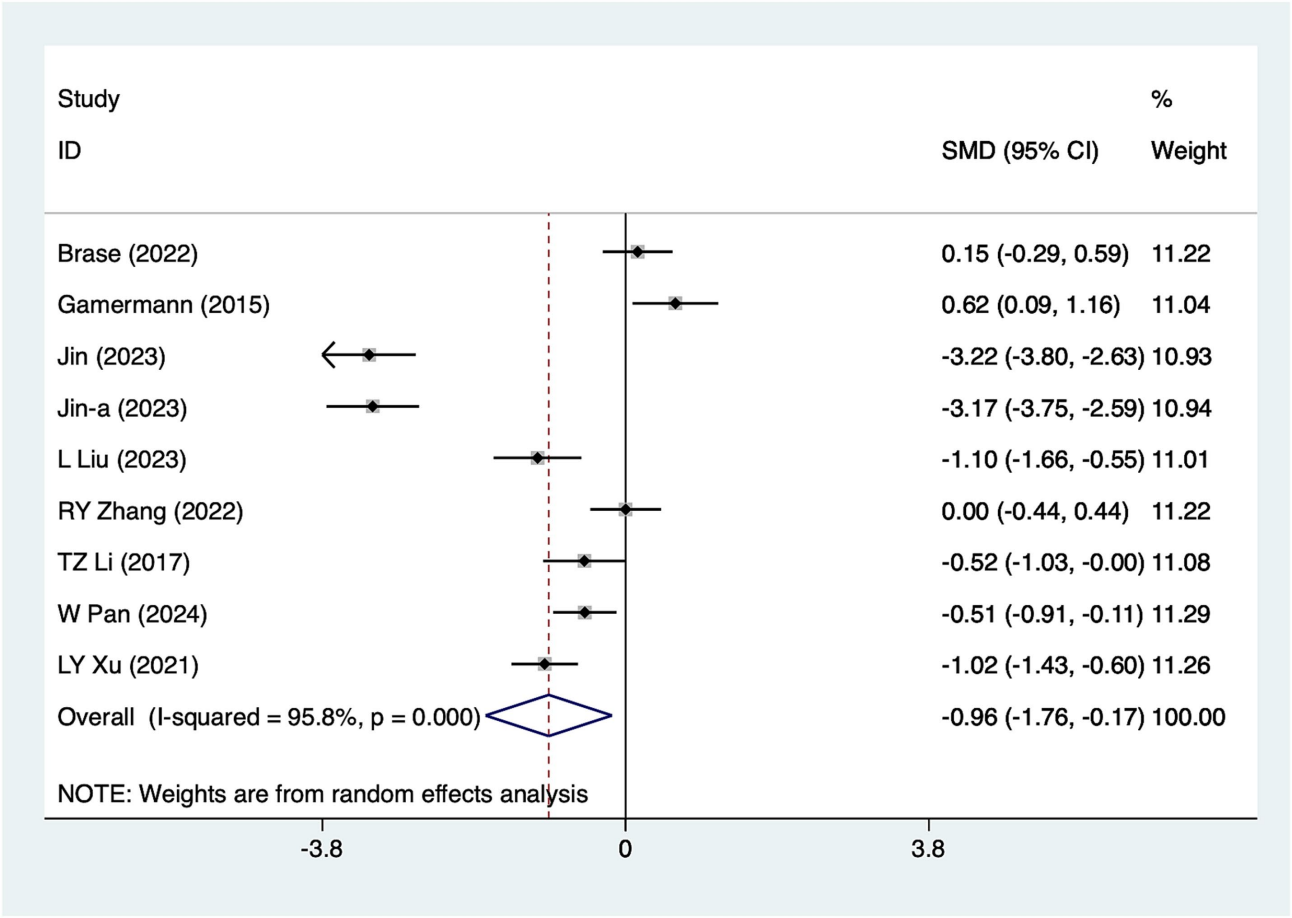
A 48-h pain score meta-analysis forest plot. The coordinate value corresponding to the box represents the difference in response rate between the experimental group and the control group for each study. The value range covered by the horizontal line represents the confidence interval for each study. The bottom diamond represents the aggregated effect.

Nine studies reported 48-h pain scores. Due to substantial heterogeneity (*I*^2^ = 95.8%, *p* = 0.001), a random-effects model was used for analysis. The results (Figure 5) suggested that acupuncture effectively reduced 48-h pain scores [SMD = -0.96, 95%CI (−1.76, −0.17)]. Subgroup analyses based on different acupuncture modalities (Table 2) showed that both electroacupuncture and conventional acupuncture were effective in reducing 48-h pain scores. Due to considerable heterogeneity, sensitivity analyses were conducted using a leave-one-out (literature-by-exclusion) approach (Supplementary Figure S6). The results indicated minimal sensitivity, suggesting that the analyses were robust and stable.

#### Bowel sound recovery time

**Figure 6:**
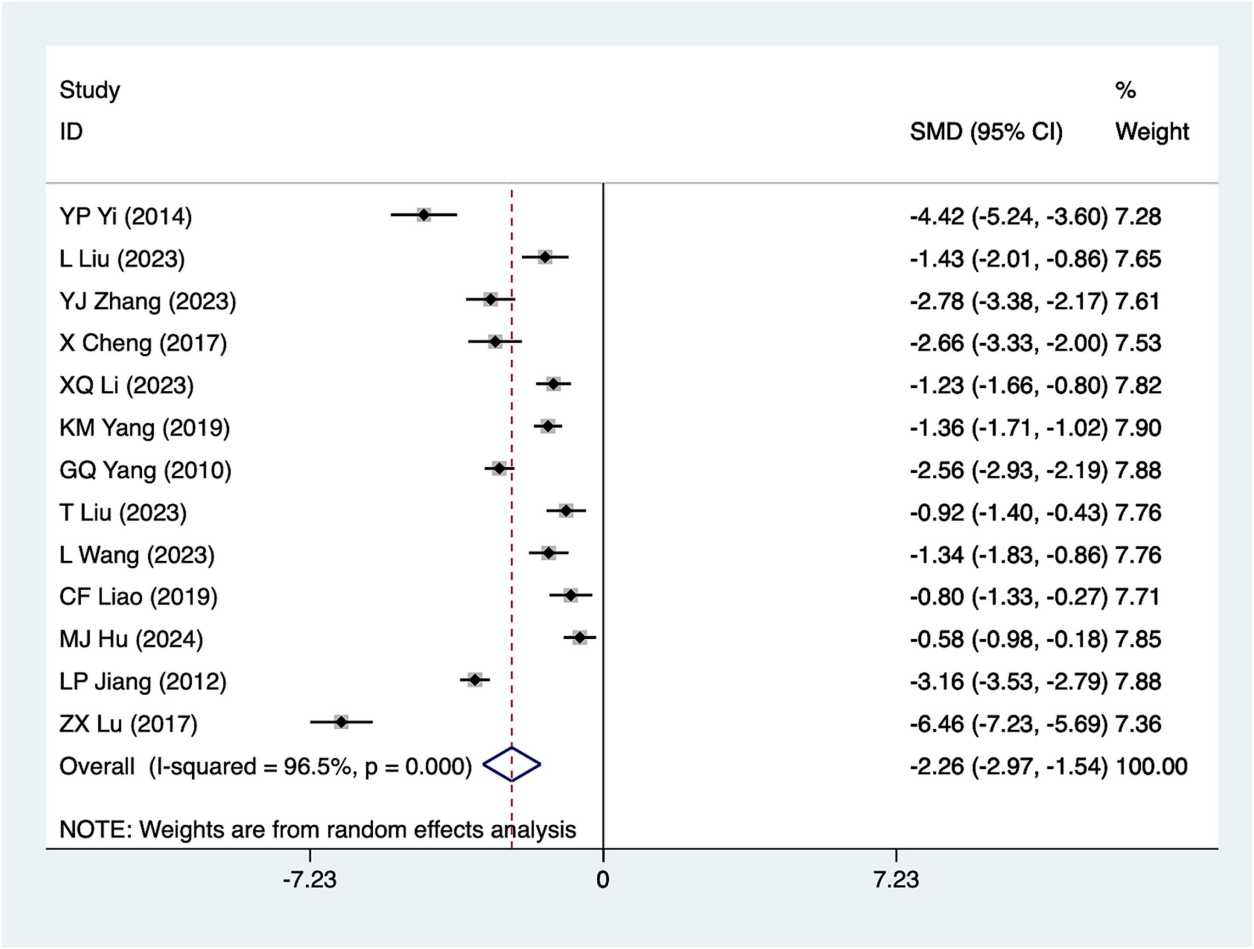
Meta-analysis forest plot of bowel sound recovery time. The coordinate value of the box represents the difference in response rate between the experimental group and the control group for each study. The value range covered by the horizontal line represents the confidence interval for each study. The bottom diamond represents the aggregated effect.

A total of 13 studies reported bowel sound recovery time. Due to significant heterogeneity (*I*^2^ = 96.5%, *p* = 0.001), a random-effects model was used for analysis. The results (Figure 6) suggested that acupuncture significantly reduced bowel sound recovery time [SMD = –2.26, 95%CI (−2.97, −1.54)]. Subgroup analyses were conducted according to different acupuncture modalities, and the results of the analyses (Table 2) showed that ordinary acupuncture was effective in reducing the recovery time of bowel sounds. Due to considerable heterogeneity, sensitivity analyses were conducted using a leave-one-out (literature-by-exclusion) approach (Supplementary Figure S7). The results indicated minimal sensitivity, suggesting that the analyses were robust and stable. Due to the presence of three high-risk studies for this metric, the results after the removal of high-risk studies (31, 43, 44) (Supplementary Figure S8) suggest that the effect sizes remained largely consistent with those before the removal of these studies [SMD = −2.28, 95% CI (−3.11, −1.46)].

#### Anal exhaust time

**Figure 7:**
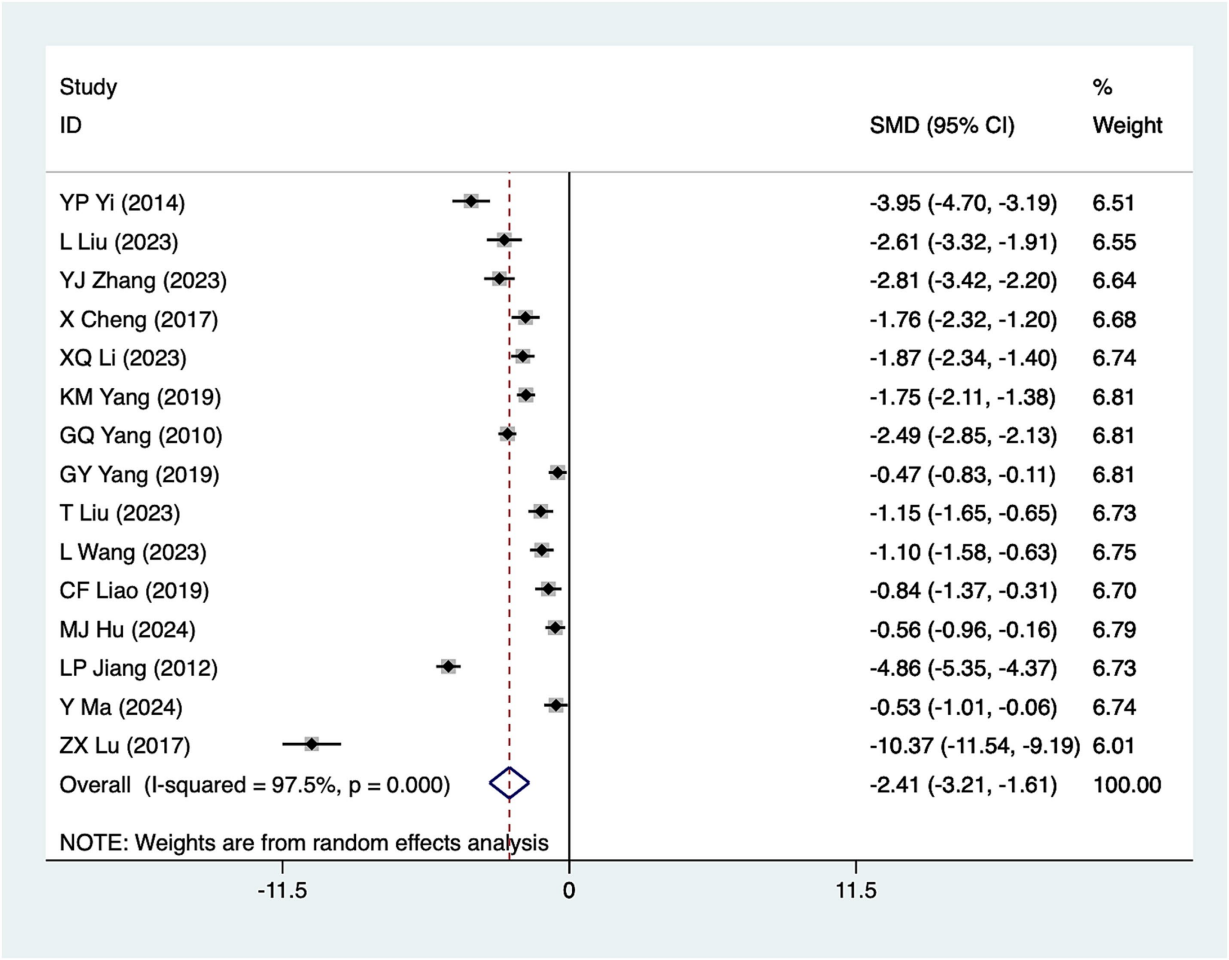
A meta-analysis forest plot of anal exhaust time. The coordinate value corresponding to the box represents the difference in response rate between the experimental group and the control group for each study. The value range covered by the horizontal line represents the confidence interval for each study. The bottom diamond represents the aggregated effect.

A total of 15 studies reported anal exhaust time. Due to significant heterogeneity (*I*^2^ = 97.5%, *p* = 0.001), a random-effects model was used for analysis. The results (Figure 7) suggested that acupuncture significantly reduced anal exhaust time [SMD = −2.41, 95% CI (−3.21, −1.61)]. Subgroup analyses based on different acupuncture modalities (Table 2) showed that conventional acupuncture was effective in reducing anal exhaust time. Due to the considerable heterogeneity, sensitivity analyses were conducted using a leave-one-out (literature-by-exclusion) approach (Supplementary Figure S9). The results suggested minimal sensitivity. Additionally, after excluding three high-risk studies (31, 43, 44), the effect size remained largely consistent (Supplementary Figure S10) [SMD = −2.31, 95% CI (−3.12, −1.50)], indicating that the analyses were robust and stable.

#### Meta-regression results

Meta-regression results (Table 3) showed that the type of intervention had a significant effect on several outcome indicators, particularly pain scores, bowel sound recovery time, and anal defecation time at 12, 24, and 48 h postoperatively—all of which demonstrated statistical significance (*p* < 0.05). These findings suggest that variations in acupuncture type may contribute to heterogeneity in these outcomes. In contrast, the year of publication was not significantly associated with any outcome, indicating that the timing of the study had a minimal impact on the results.

**Table 3:** Meta-regression results.

Outcomes	Variables	Coef.	Std. Err.	*P*	95%CI
6 h-pains scores	Year	−0.172	0.252	0.544	−0.973, 0.630
	Type	0.349	0.415	0.463	−0.973, 1.671
12 h-pains scores	Year	−0.158	0.137	0.292	−0.494, 0.177
	Type	−1.364	0.482	0.030	−2.543, −0.186
24 h-pains scores	Year	−0.055	0.058	0.366	−0.182, 0.072
	Type	−1.352	0.476	0.015	−2.388, −0.316
48 h-pains scores	Year	−0.226	0.146	0.166	−0.571, 0.119
	Type	−1.798	0.705	0.038	−3.463, −0.131
Bowel sound recovery time	Year	0.191	0.088	0.054	−0.004, 0.385
	Type	−2.683	1.078	0.030	−5.056, −0.311
Anal exhaust time	Year	0.234	0.136	0.109	−0.060, 0.528
	Type	−4.015	1.634	0.029	−7.545, −0.484

#### Publication bias

In the present study, the publication bias was assessed using the Egger test. The results indicated no evidence of publication bias for 6-h pain scores (*p* = 0.795), 12-h pain scores (*p* = 0.947), 24-h pain scores (*p* = 0.329), 48-h pain scores (*p* = 0.126), and bowel sound recovery time (*p* = 0.145). However, the test suggested a higher likelihood of publication bias for anal exhaust time (*p* = 0.018).

## Discussion

To the best of our knowledge, this is the first meta-analysis to explore the effects of acupuncture on post-cesarean section pain and gastrointestinal function. This study synthesizes 26 articles and concludes that acupuncture can reduce post-cesarean pain scores, shorten the time to bowel sound recovery, and decrease the time until the first passage of flatus.

In this study, we systematically analyzed pain scores at four time points: 6, 12, 24, and 48 h. We found that acupuncture treatment significantly reduced pain at all time points, with a particularly pronounced effect on the reduction of pain scores at 12 and 24 h. This finding supports the effectiveness of acupuncture in acute pain management and provides strong evidence for clinical pain relief. However, there was also a considerable degree of heterogeneity in the study, particularly in the analyses of the 6- and 12-h pain scores. We validated the results with subgroup analyses and sensitivity analyses to ensure the stability of the findings. Acupuncture can reduce pain by stimulating specific acupuncture points, possibly by modulating the nervous system and activating endogenous analgesic mechanisms (48). For example, acupuncture stimulates the secretion of neurotransmitters in the spinal cord and brainstem, such as endorphins, serotonin, and norepinephrine, which help inhibit the transmission of pain signals (49).

Additionally, acupuncture’s modulatory effects on the sympathetic nervous system may also play a significant role in reducing acute pain. By modulating the sensitivity of pain centers and peripheral nerves, acupuncture can effectively reduce pain perception. For pain scores at different time points, the possible mechanism is that acupuncture gradually exerts a sustained analgesic effect by modulating self-repair processes and immune responses in the body (17). For example, acupuncture showed a more significant effect in the 12- and 24-h pain scores, possibly due to its long-lasting effects on neural adaptations and pain signal processing capacity.

Subgroup analyses revealed that electroacupuncture was only weakly effective in reducing pain scores at 6 and 12 h but failed to demonstrate a significant effect in the 6-h pain score outcome. This may be related to the stimulation mode and frequency of electroacupuncture, which can produce a rapid neural response in the short term through electrical current stimulation; however, it may take a longer time to show its effect on acute pain relief (50). In contrast, conventional acupuncture may gradually produce more significant long-term relief of pain through a gentler stimulatory effect. For both 24-h and 48-h pain scores, electroacupuncture and conventional acupuncture modalities showed significant efficacy, which may reflect the similarity in long-term analgesic effects between these two acupuncture methods. The study suggests that both electroacupuncture and conventional acupuncture, despite differences in stimulation intensity and underlying mechanisms, are effective in modulating neural pathways and endocrine responses to control pain. Therefore, both may exert sustained effects on long-term pain relief (51).

In contrast to other related studies, the results of this study are consistent with the existing literature’s findings regarding the effectiveness of acupuncture for relieving acute pain. For example, previous systematic evaluations (52) have also found acupuncture to have a significant analgesic effect in patients with acute or subacute pain; however, comparisons of the effects of different types of acupuncture (electroacupuncture and conventional acupuncture) have not yielded consistent conclusions. Some studies (53, 54) have noted that electroacupuncture may have a more significant effect on pain reduction in the short term, but its long-term efficacy is similar to that of conventional acupuncture. Our study further confirms this and suggests that the effects of acupuncture treatment may manifest differently at different time points and when different methods are used.

In this study, we found that acupuncture significantly shortened the recovery time of bowel sounds, suggesting that it has a significant effect on promoting the recovery of intestinal function. Several studies (55) have shown that acupuncture can effectively promote the recovery of bowel sounds, particularly in postoperative bowel function. Acupuncture can significantly shorten the recovery time of bowel sounds and improve bowel function. Acupuncture can regulate the activity of the autonomic nervous system, especially the parasympathetic nerves, by stimulating specific acupoints. Activation of the parasympathetic nerves promotes peristalsis and the secretion of digestive juices in the intestines, thereby accelerating the recovery of intestinal function (56). The role of the parasympathetic nerves is particularly critical in the recovery of bowel sounds, as they promote the appearance of bowel sounds by modulating the activity of the smooth muscles in the intestinal wall and increasing bowel motility. In addition, acupuncture may further support the recovery of intestinal function by enhancing blood circulation to the intestines and increasing the supply of oxygen and nutrients to the intestines (57). Certain studies have also indicated that acupuncture can inhibit the accumulation of toxins in the intestines by reducing the inflammatory response, which may also positively affect the recovery of bowel sounds (58). In subgroup analyses, conventional acupuncture showed a significant effect, whereas electroacupuncture had a smaller effect on the time to recovery of bowel sounds. This finding may be related to the difference in the mechanism of action between the two. Conventional acupuncture can gradually regulate intestinal function and promote the recovery of bowel sounds through a gentler stimulating effect. Electroacupuncture, on the other hand, stimulates acupoints by means of electrical currents. Although this type of stimulation can enhance the response of the nervous system within a short period, it may not have as long-lasting a modulating effect on intestinal function as conventional acupuncture (59). This also suggests that conventional acupuncture may be more suitable for treating bowel sound recovery, especially in cases where long-term, sustained regulation of bowel function is necessary.

Anal gas expulsion is a crucial indicator of intestinal function recovery and is closely related to peristalsis, the ability to expel gas, and nerve function recovery. Acupuncture helps promote intestinal peristalsis and accelerates anal gas expulsion by modulating the activity of the autonomic nervous system, particularly the parasympathetic nerves. It may shorten the time to first anal gas expulsion by improving intestinal blood circulation, reducing intestinal inflammatory responses, and enhancing intestinal function through nerve reflexes (60). Subgroup analyses showed that conventional acupuncture was able to significantly shorten anal gas expulsion time, whereas the effect of electroacupuncture was more limited. Conventional acupuncture, which regulates acupoints through manual manipulation or low-frequency electrical stimulation, can gently but consistently regulate intestinal function and may be more suitable for long-term intestinal recovery.

In contrast, electroacupuncture produces a more direct and rapid effect through electrical current stimulation, which may be more suitable for short-term neuromodulation but has a less sustained effect than conventional acupuncture in the complex physiological process of anal gas expulsion. The study’s results showed a significant improvement in anal voiding time; however, Egger’s test identified a potential publication bias for this outcome (*p* = 0.018). This suggests that studies with better outcomes may be overrepresented in the literature. The presence of publication bias may affect the generalizability and accuracy of this finding. To mitigate this issue, a sensitivity analysis was conducted to exclude potentially biased studies. The results showed a consistent effect on anal voiding time; however, the findings should be interpreted with caution due to the presence of identified bias.

### Strengths and limitations

The strength of this study lies in the integration of several high-quality RCTs through a large-scale meta-analysis, which provides evidence of the effectiveness of acupuncture in promoting anal gas drainage and intestinal function recovery, and enhances the stability of the findings through sensitivity analyses. Subgroup analyses also explored the efficacy of different acupuncture modalities, providing more specific guidance for clinical treatment. Additionally, the study’s methodology was rigorous, which helped reduce bias and enhance the reliability of the conclusions.

Although this study yields valuable findings, it has some limitations that may impact the broad applicability of its results. First, there was a considerable amount of heterogeneity in this study, especially in the analysis of metrics such as time to postoperative exhaustion. The cause of this heterogeneity may be related to differences in study designs, patient populations, and treatment protocols. Second, the quality of some of the included studies varied, with significant differences in patient backgrounds and non-uniform treatment protocols, which may have impacted the accuracy and generalizability of the results. Given these limitations, future studies should focus more on optimizing study design, standardizing treatment protocols, and strictly controlling potential confounders. This will enhance not only the internal validity of the studies but also yield more consistent results, thereby offering more reliable evidence to support clinical practice. Further studies should focus on the specific mechanisms of action of different types of acupuncture on postoperative recovery and consider a more diverse patient population, especially patients of different ages, genders, and underlying diseases, to analyze their different responses to the efficacy of acupuncture; at the same time, prospective, randomized controlled trials should be designed to minimize bias in study design and to improve the scientific validity and clinical applicability of the findings. By strengthening the exploration of these aspects, future studies are expected to provide healthcare practitioners with more instructive conclusions to help optimize the clinical application of acupuncture treatment and enhance patients’ postoperative recovery.

### Clinical implications

The results of this meta-analysis showed that acupuncture had significant efficacy in postoperative analgesia, especially at 12, 24, and 48 h postoperatively, with electroacupuncture having a more significant analgesic effect at 12 and 48 h postoperatively. This suggests that acupuncture can be used as an effective adjunct to early postoperative analgesic management, especially in reducing pain during the critical postoperative period, which is of significant clinical value. Additionally, acupuncture can help accelerate the recovery of gastrointestinal function. The results of this study showed that acupuncture can significantly shorten the recovery time of bowel sounds and the time of defecation, suggesting that acupuncture can be used as an effective treatment to promote the recovery of gastrointestinal function in the postoperative period, and it provides a useful adjunctive measure for patients’ postoperative recovery. However, this study also found significant heterogeneity in the efficacy of different acupuncture modalities, suggesting that the choice of acupuncture modality may critically influence clinical outcomes. Therefore, when applying it clinically, medical professionals should select the appropriate acupuncture modality according to the patient’s specific condition to maximize the therapeutic effect. Finally, although the results of the sensitivity analysis yielded more stable conclusions, publication bias regarding the time of exhaustion may affect the accuracy of the results. Therefore, more high-quality studies should be conducted in the future to verify the effectiveness of acupuncture in postoperative recovery and to ensure its reliability in clinical practice.

## Conclusion

This study demonstrated the significant efficacy of acupuncture in reducing pain and promoting the recovery of bowel function. The analyses showed that acupuncture was effective in reducing pain scores at 6, 12, 24, and 48 h, postoperatively, and significantly shortening the time to bowel sound recovery and anal gas expulsion. Subgroup analyses revealed that conventional acupuncture demonstrated significant efficacy across the majority of indicators, whereas electroacupuncture was relatively ineffective at certain time points. Although the study results highlight the potential of acupuncture in enhancing postoperative recovery, the generalizability and accuracy of the findings are somewhat compromised by study heterogeneity, variations in study quality, differences in patient populations, and inconsistencies in acupuncture treatment protocols.

## Data Availability

The original contributions presented in the study are included in the article/Supplementary material, further inquiries can be directed to the corresponding author.
