# Supplementary material for: Acupuncture for post-cesarean pain and gastrointestinal function recovery: a meta-analysis and systematic review
Source: Front Med (Lausanne). 2025 Jun 18;12:1583898. doi: 10.3389/fmed.2025.1583898 (PMC12213687; doi:10.3389/fmed.2025.1583898)
Supplement: Supplementary file 1 [file Supplementary_file_1.docx]

| Study | Year | Sample size | | Mean age (years) | | ASA score | Pregnancy times | Comorbidity | Intervention | | Frequency | Duration of each treatment | outcomes |
| --- | --- | --- | --- | --- | --- | --- | --- | --- | --- | --- | --- | --- | --- |
|  |  | EG | CG | EG | CG |  |  |  | EG | CG |  |  |  |
| Brase | 2022 | 38 | 42 | 32 | 32 | ASA I-III | Primipara/ Multipara | NR | Laser acupuncture: 830 nm | Placebo | Twice/day | 30min | F1; F2 |
| Gamesman | 2015 | 28 | 28 | 28 | 26.9 | ASAI/III | Primipara/ Multipara | NR | Acupuncture: P6 and LI4 | Placebo | Twice/day | 30min | F1 |
| Jin | 2023 | 106 | 52 | 30.45 | 31.19 | NR | NR | NR | Electroacupuncture: 2/20/100HZ | Placebo | Three times/ day | 20min | F1; F3 |
| Mazda | 2018 | 20 | 18 | 34.3 | 33.7 | NR | NR | None | Acupuncture: P6 and LI4 | Placebo | Three times/ day | 20min | F1 |
| Usichenko | 2022 | 60 | 60 | 31 | 31 | ASAII/III | Primipara/ Multipara | None | Acupuncture: LI4 | Placebo | Once/day | 20min | F1; F2 |
| WU | 2009 | 40 | 20 | 30.9 | 30.8 | ASAI/II | Primipara | None | Acupuncture: LI4; Electroacupuncture: 2HZ | Placebo | Once/day | 15min | F1 |
| YP Yi | 2014 | 40 | 32 | 25.2 | 26.5 | ASAI/II | NR | NR | Acupuncture: LI4+TCM | Placebo | Three times/ day | 30min | F4; F5; F6 |
| Y He | 2022 | 52 | 52 | 28.36 | 27.42 | NR | NR | NR | Acupuncture: LI4 | Placebo | NR | 20min | F1 |
| L Liu | 2023 | 30 | 30 | 29.4 | 30.3 | NR | NR | NR | prick needling+TCM | Placebo | Once/day | NR | F1; F5; F6 |
| YJ Zhang | 2023 | 42 | 42 | 31.22 | 31.88 | NR | NR | NR | Electroacupuncture+TCM | Placebo | Twice/day | 30min | F5; F6 |
| RY Zhang | 2022 | 35 | 44 | 30.7 | 30.7 | ASAI/II | NR | NR | prick needling | Placebo |  |  | F1 |
| X Cheng | 2017 | 34 | 34 | 27.4 | 27.7 | NR | NR | NR | Warm acupuncture | Placebo | Twice/day | NR | F5; F6 |
| TZ Li | 2017 | 30 | 30 | 30 | 32 | ASAI/II | NR | NR | Electroacupuncture: 2HZ | Placebo | Twice/day | NR | F1 |
| XQ Li | 2023 | 50 | 50 | 26.46 | 26.69 | NR | NR | NR | prick needling | Placebo | Twice/day | NR | F1; F5; F6 |
| KM Yang | 2019 | 80 | 80 | 30.9 | 31.2 | NR | NR | NR | Acupuncture: P6 and LI4 | Placebo | NR | 20min | F5; F6 |
| GQ Yang | 2010 | 110 | 110 | 25.8 | 25.8 | NR | NR | NR | Acupuncture: LI4+TCM | Placebo | NR | 20min | F5; F6 |
| GY Yang | 2019 | 60 | 60 | 28 | 28 | NR | NR | NR | Auricular acupuncture | Placebo | NR | 20min | F6 |
| T Liu | 2023 | 36 | 36 | 28.45 | 28.32 | NR | NR | NR | Auricular acupuncture | Placebo | NR | 30min | F5; F6 |
| W Pan | 2024 | 50 | 50 | 25.84 | 25.61 | NR | NR | NR | Pestle acupuncture | Placebo | NR | 30min | F1 |
| L Wang | 2023 | 40 | 40 | 27.48 | 38.01 | NR | NR | NR | Pestle acupuncture | Placebo | NR | 30min | F5; F6 |
| CF Liao | 2019 | 29 | 31 | 25.22 | 25.69 | NR | Primipara/ Multipara | None | Electroacupuncture: 2HZ | Placebo | NR | 30min | F5; F6 |
| MJ Hu | 2024 | 50 | 50 | 31.6 | 31.2 | NR | Primipara/ Multipara | None | prick needling | Placebo | Twice/day | 20min | F5; F6 |
| LP Jiang | 2012 | 130 | 126 | 28 | 27 | NR | NR | NR | Electroacupuncture: 2HZ | Placebo | Twice/day | 20min | F5; F6 |
| LY Xu | 2021 | 50 | 50 | 27.48 | 27.64 | ASAI/II | NR | NR | Electroacupuncture: 2HZ | Placebo | Twice/day | 45min | F1 |
| Y Ma | 2024 | 35 | 35 | 29.51 | 30.46 | NR | NR | NR | Acupuncture: P6 and LI4 | Placebo | Twice/day | 45min | F6 |
| ZX Lu | 2017 | 82 | 82 | 23-44 | | ASAI/II | NR | NR | Warm acupuncture | Placebo | Twice/day | 30min | F5; F6 |

EG: experiment group; CG: control group; ASA: American Society of Anesthesiologists; NR: not report; TCM: Traditional Chinese medicine; F1: pain scores; F2: Length of stay; F3: Fentanyl consumption; F4: Time for bloating to disappear; F5: Bowel sound recovery time; F6: Anal exhaust time
