# Supplementary material for: Acupuncture for post-cesarean pain and gastrointestinal function recovery: a meta-analysis and systematic review
Source: Front Med (Lausanne). 2025 Jun 18;12:1583898. doi: 10.3389/fmed.2025.1583898 (PMC12213687; doi:10.3389/fmed.2025.1583898)
Supplement: Supplementary file 2 [file Supplementary_file_2.docx]

| Outcomes | Subgroup | No of study | Heterogeneity | | SMD (95%CI) | P |
| --- | --- | --- | --- | --- | --- | --- |
|  |  |  | I^2^(%) | P |  |  |
| 6h-pain scores | Electroacupuncture | 2 | 0 | 0.386 | -0.17(-0.44, 0.10) | 0.121 |
|  | Acupuncture | 1 | NR | NR | -0.5(-0.89, -0.11) | 0.001 |
|  | Acupuncture+TCM | 1 | NR | NR | -0.07(-0.58, 0.44) | 0.235 |
|  | Pestle acupuncture | 1 | NR | NR | -0.86(-1.27, -0.45) | 0.029 |
| 12h-pain scores | Electroacupuncture | 3 | 86.8 | 0.001 | -2.09(-2.93, -1.25) | 0.001 |
|  | Acupuncture | 3 | 87.8 | 0.0001 | -0.63(-1.45, 0.19) | 0.349 |
|  | Acupuncture+TCM | 1 | NR | NR | -0.61(-1.06, -0.15) | 0.002 |
|  | Pestle acupuncture | 1 | NR | NR | -1.10(-1.52, -0.68) | 0.010 |
| 24h-pain scores | Laser acupuncture | 1 | NR | NR | -0.11(-0.55,0.33) | 0.431 |
|  | Electroacupuncture | 5 | 79.3 | 0.001 | -0.59(-1.10, -0.08) | 0.001 |
|  | Acupuncture | 5 | 94.5 | 0.0001 | -1.94(-2.99, -0.89) | 0.031 |
|  | Acupuncture+TCM | 2 | 0 | 0.810 | -0.32(-0.66, 0.02) | 0.340 |
|  | Pestle acupuncture | 1 | NR | NR | -1.55(-2.00, -1.10) | 0.001 |
| 48h-pain scores | Laser acupuncture | 1 | NR | NR | 0.15(-0.29, 0.59) | 0.130 |
|  | Electroacupuncture | 4 | 96.3 | 0.0001 | -1.97(-3.32, -0.63) | 0.001 |
|  | Acupuncture | 1 | NR | NR | 0.62(0.09, 1.16) | 0.001 |
|  | Acupuncture+TCM | 2 | 89.3 | 0.002 | -0.54(-1.62, 0.54) | 0.230 |
|  | Pestle acupuncture | 1 | NR | NR | -0.51(-0.91, -0.11) | 0.010 |
|  | Acupuncture | 2 | 74 | 0.05 | -0.26(-0.79, 0.26) | 0.160 |
|  | Auricular acupuncture | 1 | NR | NR | -0.39(-0.75, -0.03) | 0.001 |
| Bowel sound recovery time | Acupuncture+TCM | 3 | 94.2 | 0.001 | -2.77(-4.12, -1.42) | 0.011 |
|  | Electroacupuncture + TCM | 1 | NR | NR | -2.78(-3.38, -2.17) | 0.001 |
|  | Warm acupuncture | 2 | 98.1 | 0.001 | -4.55(-8.27, -0.84) | 0.003 |
|  | Acupuncture | 3 | 78 | 0.011 | -1.06(-1.54, -0.58) | 0.001 |
|  | Auricular acupuncture | 1 | NR | NR | -0.92(-1.40, -0.43) | 0.020 |
|  | Pestle acupuncture | 1 | NR | NR | -1.34(-1.83, -0.86) | 0.001 |
|  | Electroacupuncture | 2 | 98.1 | 0.001 | -1.99(-4.30, 0.33) | 0.221 |
| Anal exhaust time | Acupuncture+TCM | 3 | 83.1 | 0.003 | -2.97(-3.82, -2.13) | 0.001 |
|  | Electroacupuncture + TCM | 1 | NR | NR | -2.81(-3.42, -2.20) | 0.021 |
|  | Warm acupuncture | 2 | 99.4 | 0.001 | -6.05(-14.48, 2.39) | 0.231 |
|  | Acupuncture | 4 | 91.0 | 0.001 | -1.18(-1.89, -0.47) | 0.002 |
|  | Auricular acupuncture | 2 | 78.3 | 0.032 | -0.78(-1.45, -0.12) | 0.034 |
|  | Pestle acupuncture | 1 | NR | NR | -1.10(-1.58, -0.63) | 0.021 |
|  | Electroacupuncture | 2 | 99.2 | 0.001 | -2.58(-6.80, 1.09) | 0.435 |
