# Supplementary material for: Acupuncture for post-cesarean pain and gastrointestinal function recovery: a meta-analysis and systematic review
Source: Front Med (Lausanne). 2025 Jun 18;12:1583898. doi: 10.3389/fmed.2025.1583898 (PMC12213687; doi:10.3389/fmed.2025.1583898)
Supplement: Supplementary file 3 [file Supplementary_file_3.docx]

| Outcomes | Variables | Coef. | Std. Err. | P | 95%CI |
| --- | --- | --- | --- | --- | --- |
| 6h-pains scores | Year | -0.172 | 0.252 | 0.544 | -0.973, 0.630 |
|  | Type | 0.349 | 0.415 | 0.463 | -0.973, 1.671 |
| 12h-pains scores | Year | -0.158 | 0.137 | 0.292 | -0.494, 0.177 |
|  | Type | -1.364 | 0.482 | 0.030 | -2.543, -0.186 |
| 24h-pains scores | Year | -0.055 | 0.058 | 0.366 | -0.182, 0.072 |
|  | Type | -1.352 | 0.476 | 0.015 | -2.388, -0.316 |
| 48h-pains scores | Year | -0.226 | 0.146 | 0.166 | -0.571, 0.119 |
|  | Type | -1.798 | 0.705 | 0.038 | -3.463, -0.131 |
| bowel sound recovery time | Year | 0.191 | 0.088 | 0.054 | -0.004, 0.385 |
|  | Type | -2.683 | 1.078 | 0.030 | -5.056, -0.311 |
| anal exhaust time | Year | 0.234 | 0.136 | 0.109 | -0.060, 0.528 |
|  | Type | -4.015 | 1.634 | 0.029 | -7.545, -0.484 |
