# Supplementary material for: Acupuncture for post-cesarean pain and gastrointestinal function recovery: a meta-analysis and systematic review
Source: Front Med (Lausanne). 2025 Jun 18;12:1583898. doi: 10.3389/fmed.2025.1583898 (PMC12213687; doi:10.3389/fmed.2025.1583898)
Supplement: Supplementary file 4 [file Supplementary_file_4.docx]

Table S1 specific search strategy

| PubMed | (((((((((("Acupuncture"[Mesh]) OR ((Acupuncture[MeSH Terms]) OR (Pharmacopuncture[Title/Abstract]))) OR ("Acupuncture Therapy"[Mesh])) OR (((((((((((Acupuncture Therapy[MeSH Terms]) OR (Acupuncture Treatment[Title/Abstract])) OR (Acupuncture Treatments[Title/Abstract])) OR (Treatment, Acupuncture[Title/Abstract])) OR (Therapy, Acupuncture[Title/Abstract])) OR (Pharmacoacupuncture Treatment[Title/Abstract])) OR (Treatment, Pharmacoacupuncture[Title/Abstract])) OR (Pharmacoacupuncture Therapy[Title/Abstract])) OR (Therapy, Pharmacoacupuncture[Title/Abstract])) OR (Acupotomy[Title/Abstract])) OR (Acupotomies[Title/Abstract]))) OR ("Acupuncture, Ear"[Mesh])) OR ((((((((Acupuncture, Ear[MeSH Terms]) OR (Acupunctures, Ear[Title/Abstract])) OR (Ear Acupunctures[Title/Abstract])) OR (Acupuncture, Auricular[Title/Abstract])) OR (Acupunctures, Auricular[Title/Abstract])) OR (Auricular Acupunctures[Title/Abstract])) OR (Auricular Acupuncture[Title/Abstract])) OR (Ear Acupuncture[Title/Abstract]))) OR ("Moxibustion"[Mesh])) OR ((Moxibustion[MeSH Terms]) OR (Moxabustion[Title/Abstract]))) OR ("Electroacupuncture"[Mesh])) OR ((((Electroacupuncture[MeSH Terms]) OR (Warm acupuncture[Title/Abstract])) OR (Dry needle[Title/Abstract])) OR (Fire needle[Title/Abstract]))) AND (("Cesarean Section"[Mesh]) OR ((((((((((((Cesarean Section[MeSH Terms]) OR (Cesarean Sections[Title/Abstract])) OR (Abdominal Delivery[Title/Abstract])) OR (C-Section (OB[Title/Abstract]))) OR (C Section (OB[Title/Abstract]))) OR (C-Sections (OB[Title/Abstract]))) OR (Caesarean Section[Title/Abstract])) OR (Caesarean Sections[Title/Abstract])) OR (Delivery, Abdominal[Title/Abstract])) OR (Abdominal Deliveries[Title/Abstract])) OR (Deliveries, Abdominal[Title/Abstract])) OR (Postcesarean Section[Title/Abstract]))) |
| --- | --- |
| Embase | \| No. \| Query \| \| --- \| --- \| \| #48 \| #33 AND #47 \| \| #47 \| #34 OR #35 OR #36 OR #37 OR #38 OR #39 OR #40 OR #41 OR #42 OR #43 OR #44 OR #45 OR #46 \| \| #46 \| 'postcesarean section':ab,ti \| \| #45 \| 'deliveries, abdominal':ab,ti \| \| #44 \| 'abdominal deliveries':ab,ti \| \| #43 \| 'delivery, abdominal':ab,ti \| \| #42 \| 'caesarean sections':ab,ti \| \| #41 \| 'caesarean section':ab,ti \| \| #40 \| 'c-sections (ob)':ab,ti \| \| #39 \| 'c section (ob)':ab,ti \| \| #38 \| 'c-section (ob)':ab,ti \| \| #37 \| 'abdominal delivery':ab,ti \| \| #36 \| 'cesarean sections':ab,ti \| \| #35 \| 'cesarean section':ab,ti \| \| #34 \| 'cesarean section'/exp \| \| #33 \| #1 OR #2 OR #3 OR #4 OR #5 OR #6 OR #7 OR #8 OR #9 OR #10 OR #11 OR #12 OR #13 OR #14 OR #15 OR #16 OR #17 OR #18 OR #19 OR #20 OR #21 OR #22 OR #23 OR #24 OR #25 OR #26 OR #27 OR #28 OR #29 OR #30 OR #31 OR #32 \| \| #32 \| 'fire needle':ab,ti \| \| #31 \| 'dry needling':ab,ti \| \| #30 \| 'warm acupuncture':ab,ti \| \| #29 \| 'electroacupuncture':ab,ti \| \| #28 \| 'electroacupuncture'/exp \| \| #27 \| 'moxabustion':ab,ti \| \| #26 \| 'moxibustion':ab,ti \| \| #25 \| 'moxibustion'/exp \| \| #24 \| 'ear acupuncture':ab,ti \| \| #23 \| 'auricular acupuncture':ab,ti \| \| #22 \| 'auricular acupunctures':ab,ti \| \| #21 \| 'acupunctures, auricular':ab,ti \| \| #20 \| 'acupuncture, auricular':ab,ti \| \| #19 \| 'ear acupunctures':ab,ti \| \| #18 \| 'acupunctures, ear':ab,ti \| \| #17 \| 'auricular acupuncture':ab,ti \| \| #16 \| 'auricular acupuncture'/exp \| \| #15 \| 'acupotomies':ab,ti \| \| #14 \| 'acupotomy':ab,ti \| \| #13 \| 'therapy, pharmacoacupuncture':ab,ti \| \| #12 \| 'pharmacoacupuncture therapy':ab,ti \| \| #11 \| 'treatment, pharmacoacupuncture':ab,ti \| \| #10 \| 'pharmacoacupuncture treatment':ab,ti \| \| #9 \| 'therapy, acupuncture':ab,ti \| \| #8 \| 'treatment, acupuncture':ab,ti \| \| #7 \| 'acupuncture treatments':ab,ti \| \| #6 \| 'acupuncture treatment':ab,ti \| \| #5 \| 'acupuncture':ab,ti \| \| #4 \| 'acupuncture'/exp \| \| #3 \| 'pharmacopuncture':ab,ti \| \| #2 \| 'acupuncture':ab,ti \| \| #1 \| 'acupuncture'/exp \| |
| Cochrane library | #1 MeSH descriptor: [Acupuncture] explode all trees 225  #2 (Acupuncture):ti,ab,kw OR (Pharmacopuncture):ti,ab,kw 21296  #3 MeSH descriptor: [Acupuncture Therapy] explode all trees 7309  #4 (Acupuncture Treatment):ti,ab,kw OR (Acupuncture Treatments):ti,ab,kw OR (Treatment, Acupuncture):ti,ab,kw OR (Therapy, Acupuncture):ti,ab,kw OR (Pharmacoacupuncture Treatment):ti,ab,kw 17204  #5 (Treatment, Pharmacoacupuncture):ti,ab,kw OR (Pharmacoacupuncture Therapy):ti,ab,kw OR (Therapy, Pharmacoacupuncture):ti,ab,kw OR (Acupotomy):ti,ab,kw OR (Acupotomies):ti,ab,kw 153  #6 MeSH descriptor: [Acupuncture, Ear] explode all trees 271  #7 (Acupunctures, Ear):ti,ab,kw OR (Ear Acupunctures):ti,ab,kw OR (Acupuncture, Auricular):ti,ab,kw OR (Acupunctures, Auricular):ti,ab,kw OR (Auricular Acupunctures):ti,ab,kw 1064  #8 (Auricular Acupuncture):ti,ab,kw OR (Ear Acupuncture):ti,ab,kw 1478  #9 MeSH descriptor: [Moxibustion] explode all trees 697  #10 (Moxabustion):ti,ab,kw 0  #11 MeSH descriptor: [Electroacupuncture] explode all trees 1207  #12 (Electroacupuncture):ti,ab,kw OR (Warm acupuncture):ti,ab,kw OR (Dry needle):ti,ab,kw OR (Fire needle):ti,ab,kw 4848  #13 #1or#2or#3or#4or#5or#6or#7or#8or#9or#10or#11or#12 23330  #14 MeSH descriptor: [Cesarean Section] explode all trees 4653  #15 (Cesarean Section):ti,ab,kw OR (Cesarean Sections):ti,ab,kw OR (Abdominal Delivery):ti,ab,kw OR (C-Section (OB)):ti,ab,kw OR (C Section (OB)):ti,ab,kw 18006  #16 (C-Sections (OB)):ti,ab,kw OR (Caesarean Section):ti,ab,kw OR (Caesarean Sections):ti,ab,kw OR (Delivery, Abdominal):ti,ab,kw OR (Abdominal Deliveries):ti,ab,kw 18020  #17 (Deliveries, Abdominal):ti,ab,kw OR (Postcesarean Section):ti,ab,kw 729  #18 #14or#15or#16or#17 18026  #19 #13and#18 204 |
| Web of science | \| 1 \| TS=(Acupuncture) OR TS=(Pharmacopuncture) OR TS=(Acupuncture Therapy) OR TS=(Acupuncture Treatment) OR TS=(Acupuncture Treatments) OR TS=(Treatment, Acupuncture) OR TS=(Therapy, Acupuncture) OR TS=(Pharmacoacupuncture Treatment) OR TS=(Treatment, Pharmacoacupuncture) OR TS=(Pharmacoacupuncture Therapy) OR TS=(Therapy, Pharmacoacupuncture) OR TS=(Acupotomy) OR TS=(Acupotomies) OR TS=(Acupuncture, Ear) OR TS=(Acupunctures, Ear) OR TS=(Ear Acupunctures) OR TS=(Acupuncture, Auricular) OR TS=(Acupunctures, Auricular) OR TS=(Auricular Acupunctures) OR TS=(Auricular Acupuncture) OR TS=(Ear Acupuncture) OR TS=(Moxibustion) OR TS=(Moxabustion) OR TS=(Electroacupuncture ) OR TS=(Warm acupuncture) OR TS=(Dry needle) OR TS=(Fire needle) \| \| --- \| --- \| \| 2 \| TS=(Cesarean Section) OR TS=(Cesarean Sections) OR TS=(Abdominal Delivery) OR TS=(C-Section (OB)) OR TS=(C Section (OB)) OR TS=(C-Sections (OB)) OR TS=(Caesarean Section) OR TS=(Caesarean Sections) OR TS=(Delivery, Abdominal) OR TS=(Abdominal Deliveries) OR TS=(Deliveries, Abdominal) OR TS=(Postcesarean Section) \| \| 3 \| #1 AND #2 \| |

Table S2 GRADE result

| Outcomes | Grade |
| --- | --- |
| 6h pain scores | Low |
| 12h pain scores | moderate |
| 24h pain scores | moderate |
| 48h pain scores | moderate |
| Bowel sound recovery time | moderate |
| Anal exhaust time | moderate |


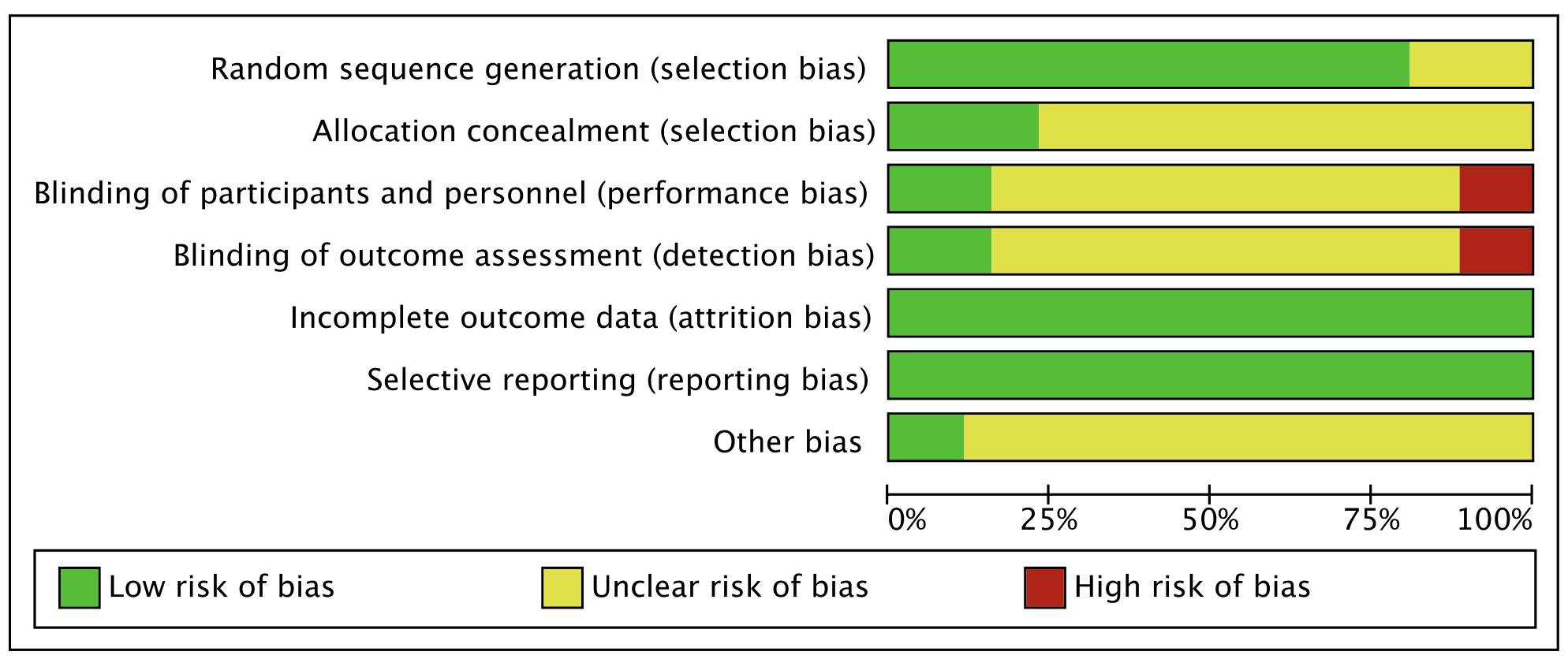


figure S1 risk of graph


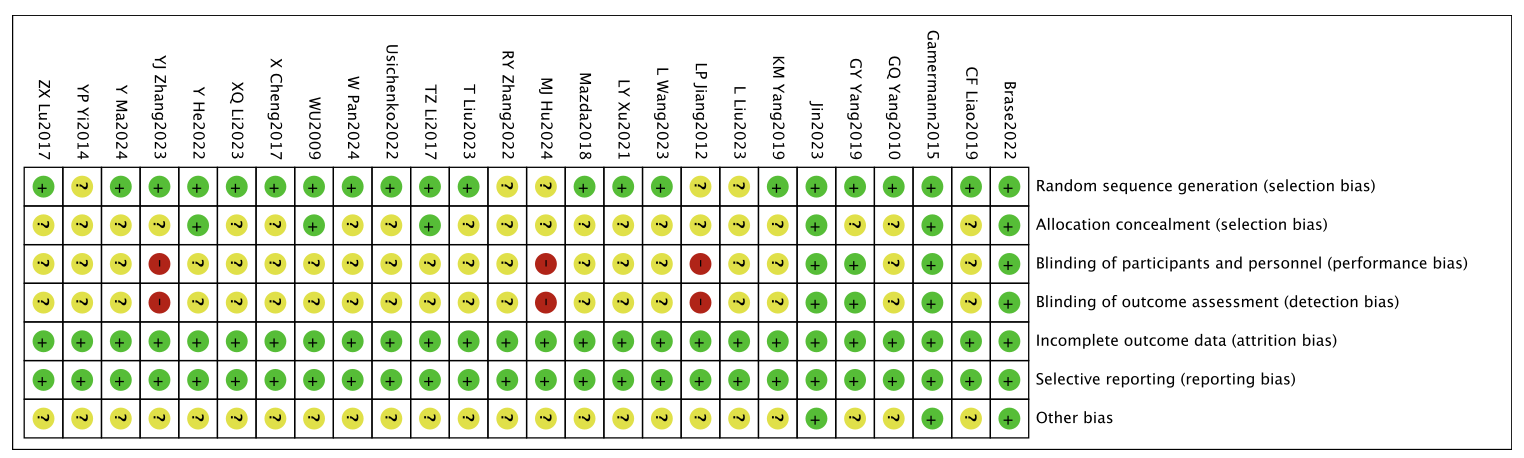


figure s2 risk of summary


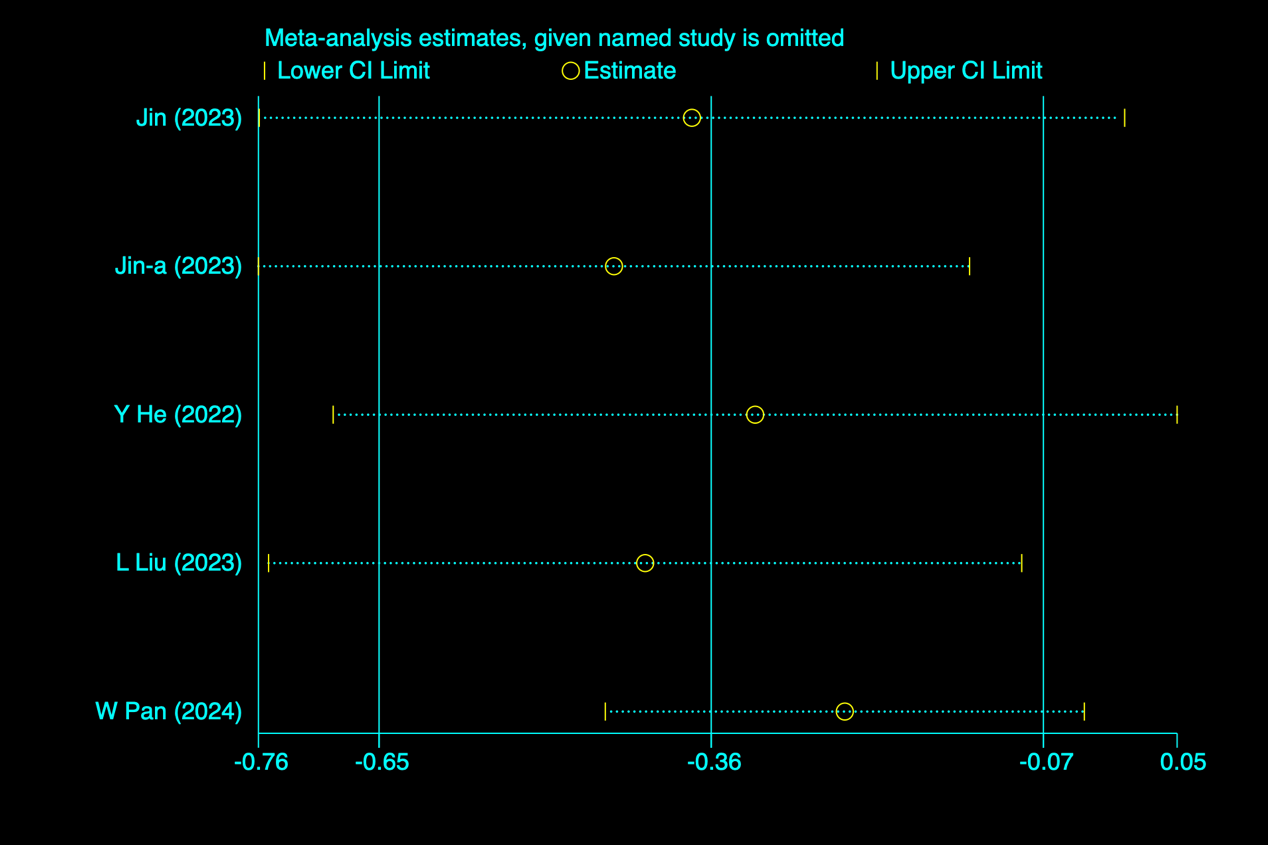


figure S3 6h pain scores Sensitivity analysis


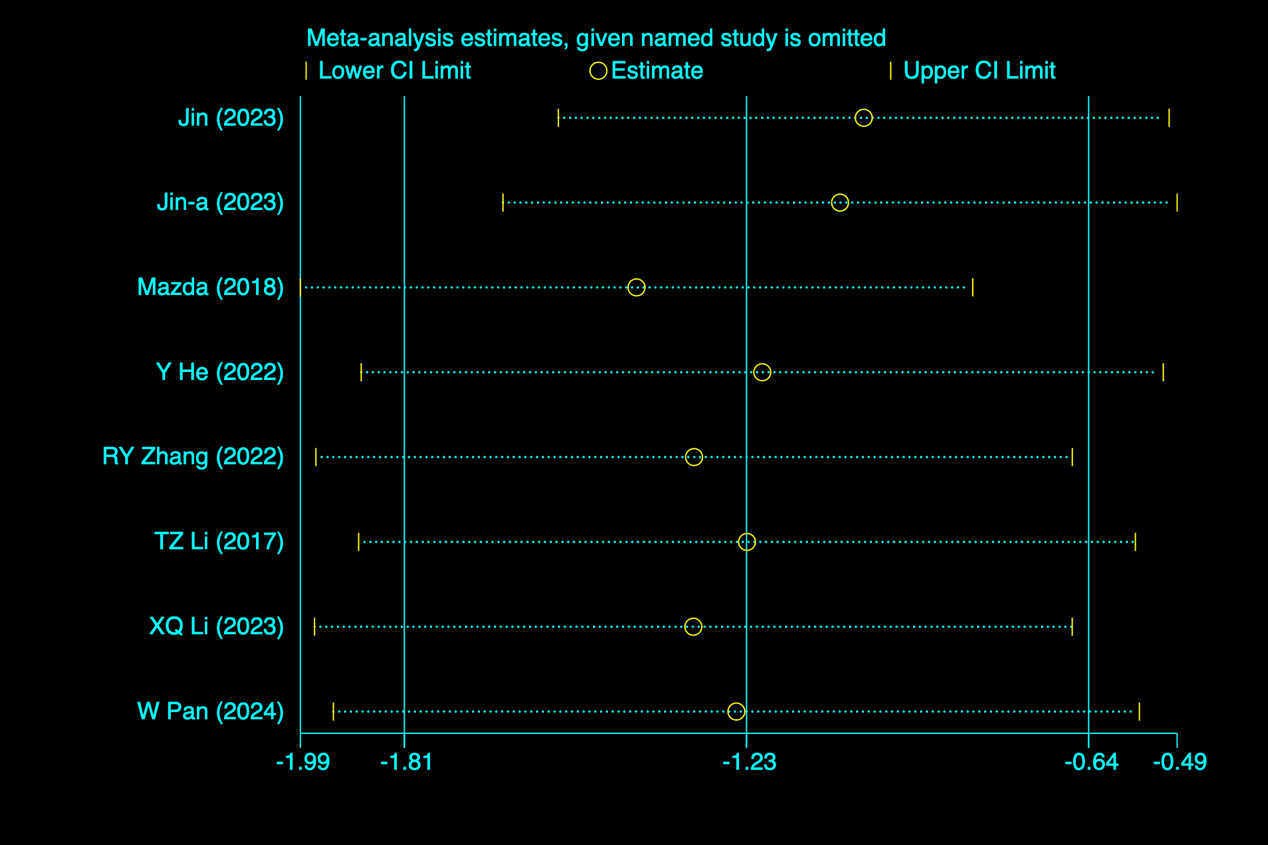


figure S4 12h pain scores Sensitivity analysis


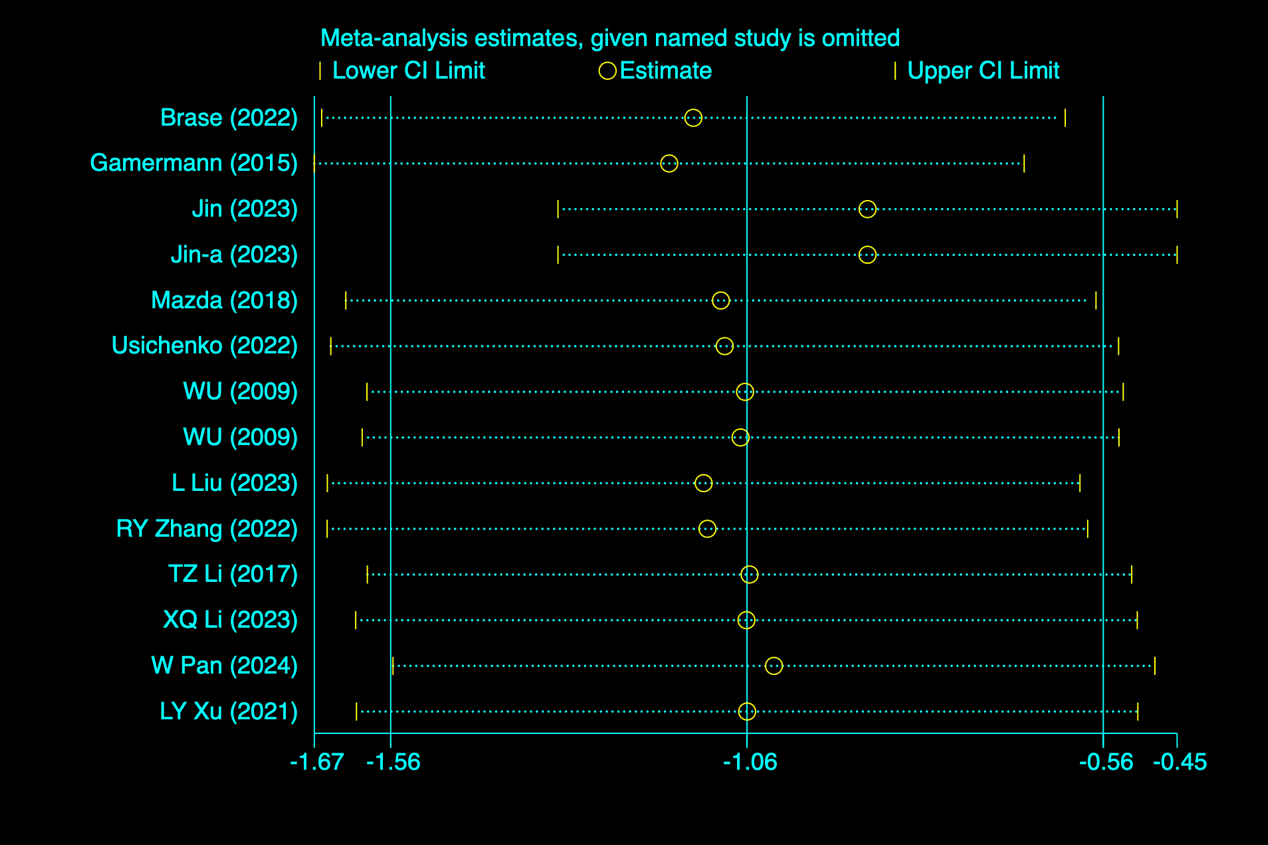


figure S5 24h pain scores Sensitivity analysis


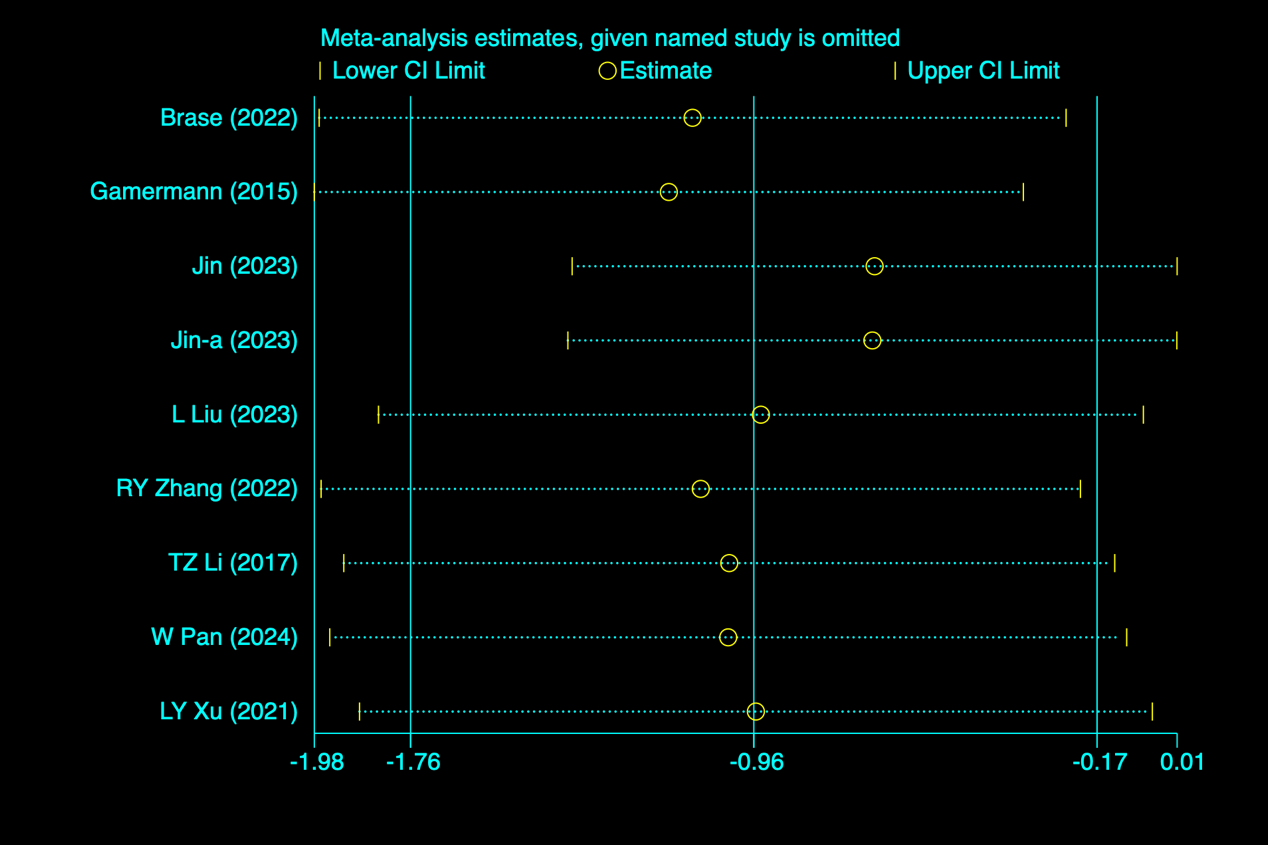


figure S6 48h pain scores Sensitivity analysis


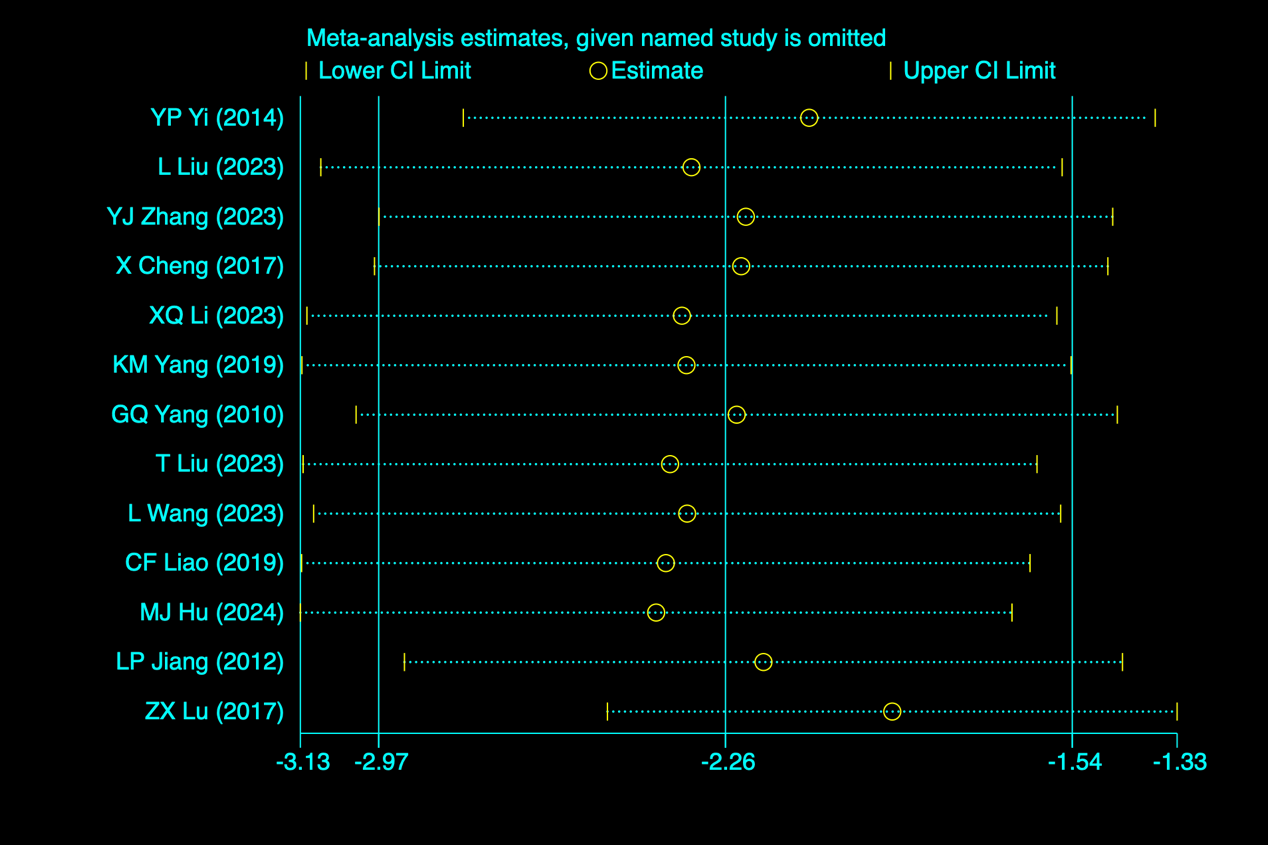


figure S7 bowel sound recovery time Sensitivity analysis


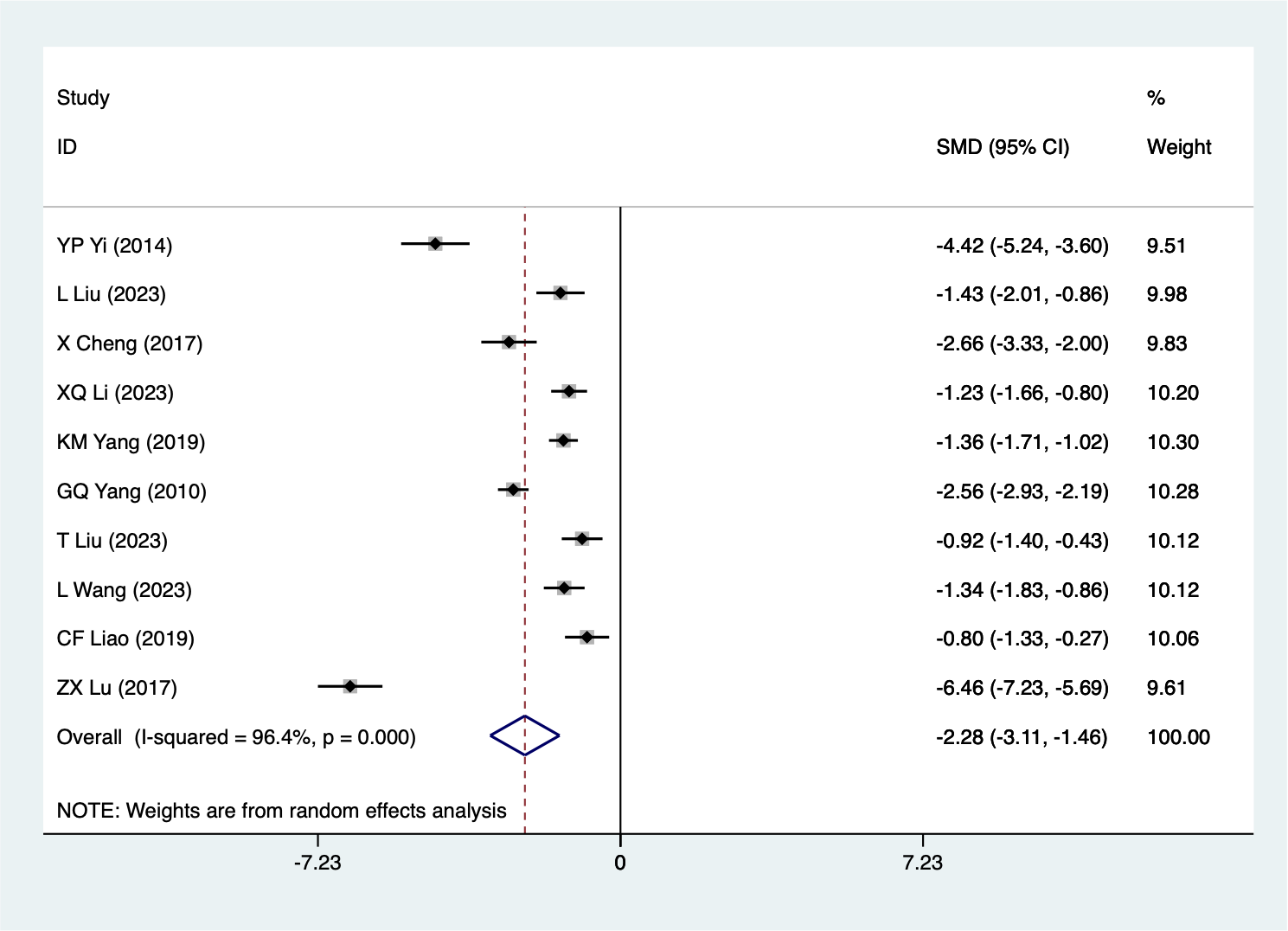


Figure S8 Meta-analysis results of Bowel sound recovery time after removing high-risk studies


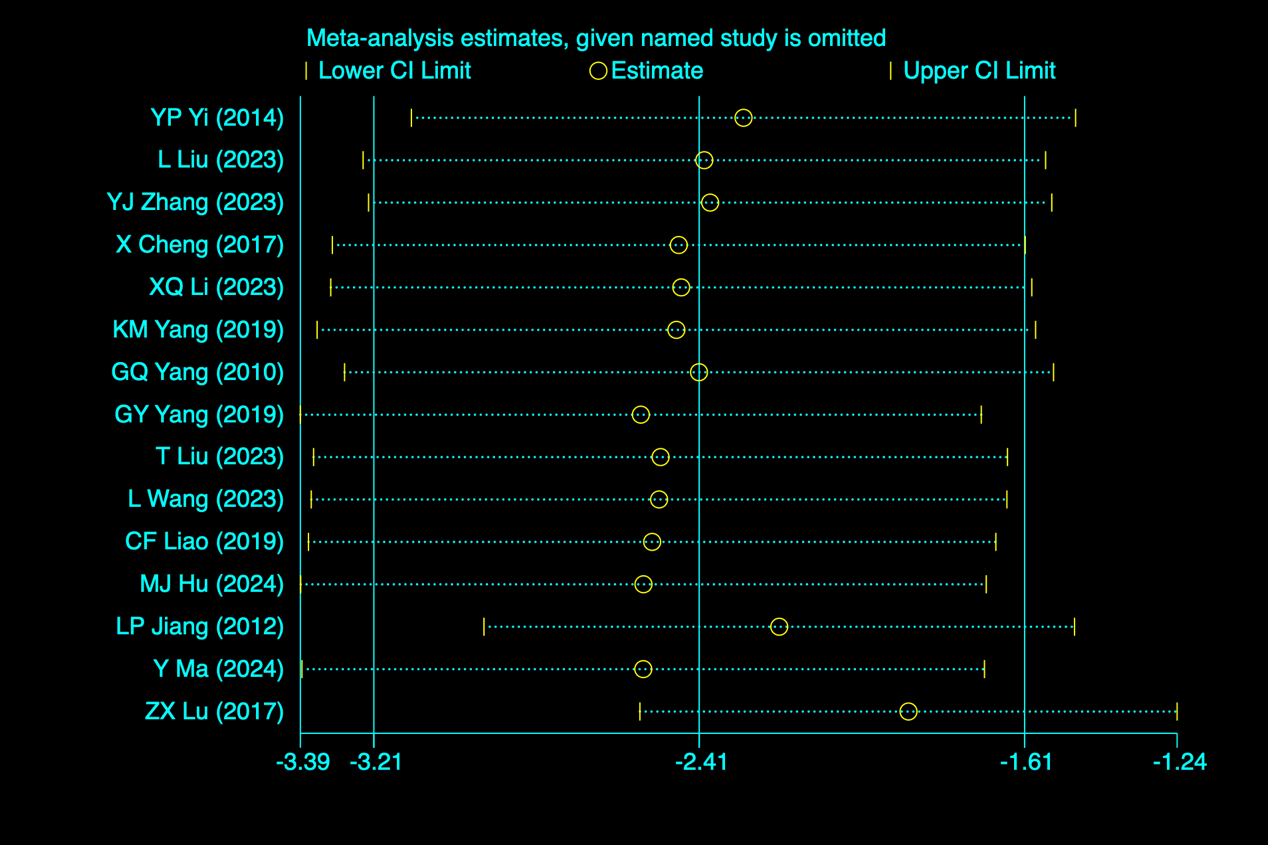


figure S9 anal exhaust time Sensitivity analysis


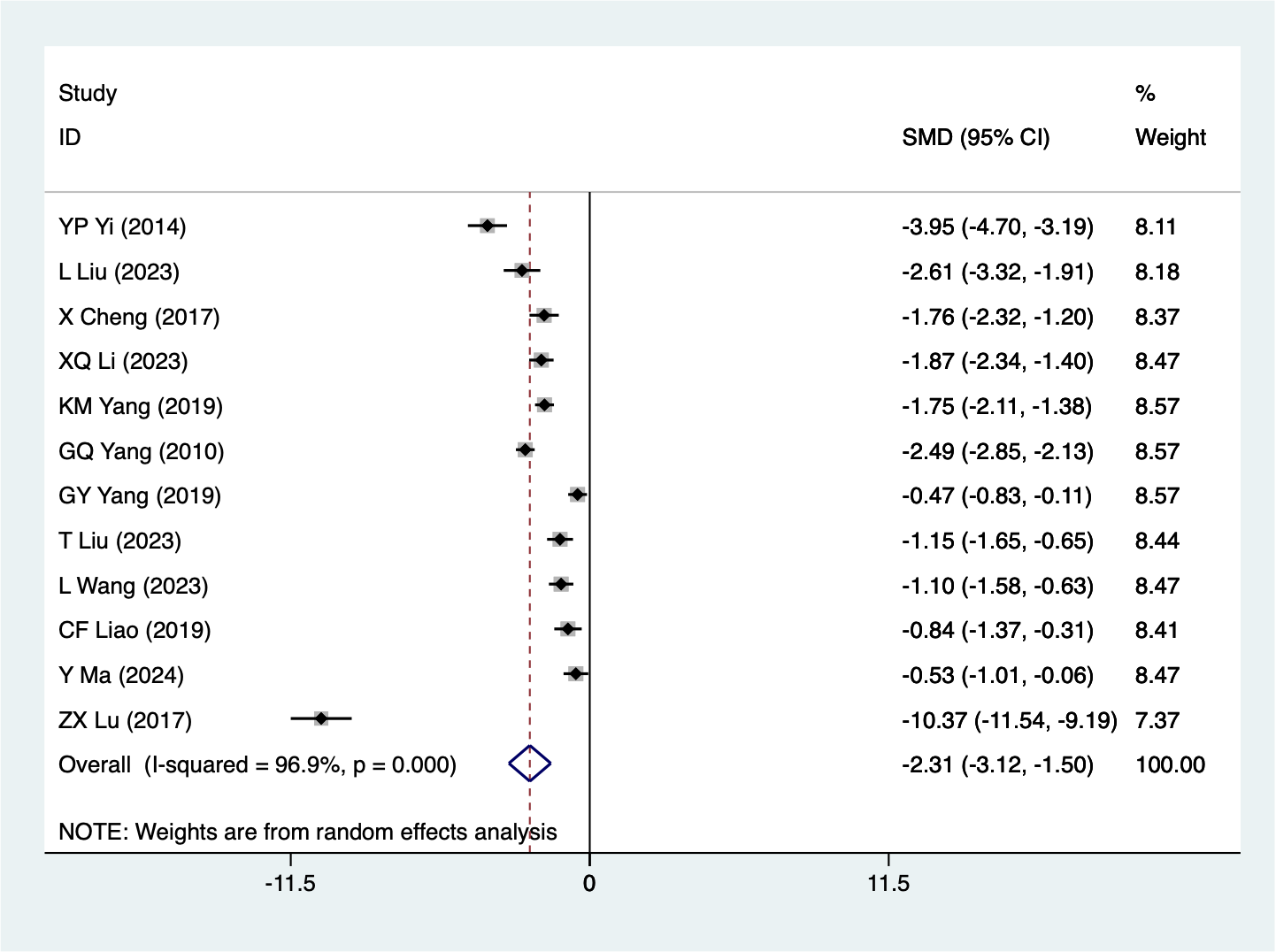


Figure S10 Meta-analysis results of Anal exhaust time after removing high-risk studies
